# PROTOCOL: Early childhood education programs for improving the development and achievement of low‐income children: a systematic review

**DOI:** 10.1002/cl2.1100

**Published:** 2020-09-02

**Authors:** Douglas J. Besharov, Douglas M. Call, Jason M. Scott

**Affiliations:** ^1^ School of Public Policy University of Maryland College Park Maryland USA

## 1 BACKGROUND

### 1.1 Description of the problem or condition

For years, the achievement gap, most commonly measured by math and reading scores, was described in terms of race. More recently, however, greater attention has been focused on differences based on family income. Under either measure, the achievement gap is large and persistent, with serious economic, social, and ethical consequences.

A large body of research finds that gaps in achievement appear well before children enter primary school (see, e.g., Bradbury, Corak, Waldfogel, & Washbrook, 2015; Burchinal et al., 2011; Ferguson, 2015; Fernals, Marchman, & Weisleder, 2013; Halle et al., 2009; Wang, 2008) and that they are substantial when children enter kindergarten (see, e.g., Brooks‐Gunn, Klebanov, Smith, Duncan, & Lee, 2003; Chapin, 2006; Chatterji, 2006; Fryer & Levitt, 2004; Lee & Burkam, 2002; Reardon & Galindo, 2009; Reardon & Portilla, 2016; Reardon & Robinson, 2012).

Whether or not there is a direct causal relationship, the achievement gap is associated with higher rates of grade retention, special education placement, school suspension and other disciplinary actions, higher school dropout rates, lower college attendance and completion rates, and lower workforce skills generally, leading to a lifetime of lower employment rates and lower average earnings (Aud, Fox, & Kewal Ramani, 2010; BLS Employment Situation, 2015a; BLS Weekly Earnings, 2015b; Hipple, 2010; Stillwell & Sable, 2013; U.S. Department of Education, 2010, 2012).

The achievement gap and its apparent consequences gain greater moment given both the changing demographics and widening income distributions being observed in the United States and elsewhere. Its continued persistence may, as Hanushek et al. (2019) warn, “spell limited intergenerational mobility in the 21st Century” (p. 1).

### 1.2 Description of the intervention

There are many policy proposals for narrowing the achievement gap, starting with more effective K‐12 education, and including expanded parental education and teen pregnancy prevention programs, increased neighborhood and school integration, and more income support and policies to decrease income inequality. But, by far, the most prominent proposed solution is expanded and enhanced early childhood education (ECE), that is, center‐based, predominantly classroom‐style programs serving preschool children.

There is, however, no set or uniform model for an ECE intervention, although most provide some form of educational programming directly to preschool children. The following are some of the key variations:

- *Age at which children begin receiving services*. Just about all programs begin by age four, with many starting at age three, and some starting before that.
- *Ancillary service(s) provided*. Some programs provide parenting skills training, some provide medical/dental services, some both, as well as other services.
- *Eligibility*. Some programs target particular groups of children (such as “at risk” children or those from families below a specified income threshold), some are neighborhood oriented, and others are available universally, regardless of income or other disadvantage.
- *Curriculum used (if any)*. Some programs use a particular curriculum (most commonly, Creative Curriculum and HighScope Curriculum), some do not specify what, if any, curriculum they use (only that each center/classroom follow one), and others implement state or locally developed curricula.
- *Focus of the program*. Some programs primarily target children's cognitive skills, some target socioemotional skills, and some focus on “whole‐child” development, or others some combination or variant thereof.
- *Follow‐on services*. Some programs provide services through the 1st year (or later) of primary school.
- *Intensity*. Some programs are full‐day, some are half‐day, and others are somewhere in between.
- *Length/duration*. Some programs are 1‐year (or more) in length, some are about 9–10 months long (or, an academic year), and, in the past, others, such as the first version of Head Start, were for shorter periods (like the summer).
- *Service setting*. Most programs are operated are in private, nonprofit centers, but many are in public schools.
- *Source of funding*. Most are state/local programs, often supported through federal funds, some are federal programs, and others are demonstration projects run by universities or other nongovernmental agencies.
- *Teacher credentials*. Some programs require that teachers have bachelor's degrees, some require associate's degrees, some require a certificate in ECE or development, some require a combination of these, and others require none.

Even among Head Start programs (which are subject to national performance standards), the variation among these and other dimensions is large. Some Head Start programs are full‐day, but most are part‐day. Some are one‐academic‐year long (usually 9‐plus months); others are longer. About half of Head Start programs use the Creative Curriculum,^1^ although about seven other major curricula are being used by the remaining programs, with none used by more than 15% (Moiduddin, Aikens, Tarullo, West, & Xue, 2012).

This variation does not appear to be unique to Head Start nor to the U.S. experience. For example, the UK's Sure Start programs provide substantial autonomy to local programs and, according to the National Evaluation of Sure Start researchers, “This has resulted in the emergence of great diversity in Sure Start local programmes” (Anning et al., 2004, p. 2).

### 1.3 How the intervention might work

To reach their potential, children need a combination of physical care, cognitive stimulation, and emotional support. Many children, especially the disadvantaged, need more developmental inputs than they receive from parents and family. For example, Hart and Risley (1995) report that, by age three, low‐income children may be exposed to as many as 30 million fewer words than their more affluent counterparts. Furthermore, the language to which disadvantaged children are exposed is typically less supportive and varied. Other researchers have reported similar results. For example, Brooks‐Gunn and Markman (2005) find that “the educated middle‐ to upper‐middle‐class 'speech culture' provides more language, more varied language, more language topics, more questions, and more conversation, all of which are linked with large vocabularies in toddlers and preschoolers” (p. 150).

The home environments of disadvantaged children often have other significant lacunae. According to a 2003 U.S. Department of Health and Human Services report, black and Hispanic children and those from families at or below the federal poverty threshold were less likely to be read to on a daily basis, to be told stories frequently, and to have visited the library at least once in the past month. A number of studies have found similar results, reporting that low‐income children have fewer books in their homes and are read to less often than their more affluent counterparts (e.g., Aikens & Barbarin, 2008; Froiland, Peterson, & Davison, 2012; Whitehurst & Lonigan, 1998; as cited in Froiland, Powell, Diamond, & Son, 2013).

Given findings such as those of Hart and Risley (1995) and Brooks‐Gunn and Markman (2005), it should not be surprising that disparities in achievement appear before children enter school.

In addition, minority and low‐income children may be more likely to experience stress, poor nutrition, and exposure to toxins, which, according to the National Scientific Council on the Developing Child (2010), may have lasting consequences for achievement. Disparities in children's early environments, therefore, might be able to explain some or much of the achievement gap at school entry. Phillips et al. (1998) estimate that these factors account for “56 percent of the math gap and 43 percent of the reading gap” observed at the end of high school.

Some research claims that brain and neurological development in a child's early years (before age four) are the indispensable foundation for social, emotional, and language skills that lead to later success. Indeed, the terms “critical periods” and “sensitive periods” are often used when discussing child development in the first 3–5 years of life (see Lombroso & Pruett, 2004, for a longer discussion.) There is no doubt that these periods are important, but the degree to which they are determinative of later life is unclear (see Bruer, 2004 and Lombroso and Pruett, 2004, for competing discussions).

In theory, then, some sort of compensatory intervention, occurring in early childhood, could narrow the developmental and longer‐term achievement gap stemming from disparities in the early home environment—by better preparing disadvantaged children for school and later life. The question for this systematic review is the degree to which research and evaluation confirms this hypothesis. As we will see, many think this is less likely because the dosage (and quality) of the intervention is not sufficient to overcome the family and environmental factors that also influence children's plasticity of learning.

#### 1.3.1 School readiness

Nearly all ECE programs attempt to increase the “school readiness” of children. Although school readiness is defined in various ways, “experts agree that readiness is a multifaceted concept that goes beyond academic and cognitive skills” (Karoly, Kilburn, & Cannon, 2005, p. 8).

The Head Start Early Learning Outcomes Framework (see Table 1) provides a detailed description of school readiness, divided into five domains:

- Approaches to learning (including behavior),
- Social and emotional development,
- Language and literacy,
- Cognition, and
- Perceptual, motor, and physical development.

**Table 1:** Head start early learning outcomes framework: domains and subdomains (infant/toddler and preschooler)

Domain	Subdomain	
	Infant/toddler	Preschooler
Approaches to learning	Emotional and behavioral self‐regulation	Emotional and behavioral self‐regulation
	Cognitive self‐regulation (executive functioning)	Cognitive self‐regulation (executive functioning)
	Initiative and curiosity	Initiative and curiosity
	Creativity	Creativity
Social and emotional development	Relationships with adults	Relationships with adults
	Relationships with other children	Relationships with other children
	Emotional functioning	Emotional functioning
	Sense of identity and belonging	Sense of identity and belonging
Language and literacy	Attending and understanding	Phonological awareness
	Communicating and speaking	Print and alphabet knowledge
	Vocabulary	Comprehension and text structure
	Emergent literacy	Writing
Cognition	Exploration and discovery	Counting and cardinality
	Memory	Operations and algebraic thinking
	Reasoning and problem‐solving	Measurement
	Emergent mathematical thinking	Geometry and spatial sense
	Imitation and symbolic representation and play	Scientific Inquiry
	–	Reasoning and problem solving
Perceptual, motor, and physical development	Perception	–
	Gross motor	Gross motor
	Fine motor	Fine motor
	Health, safety, and nutrition	Health, safety, and nutrition

*Source:* U.S. Department of Health and Human Services, Administration for Children and Families (2015), *Head Start Early Learning Outcomes Framework: Ages birth to five*, https://eclkc.ohs.acf.hhs.gov/sites/default/files/pdf/elof-ohs-framework.pdf

For each domain, a group of subdomains is provided, each comprising a set of goals and age‐appropriate milestones/benchmarks for children from birth‐to‐five.

#### 1.3.2 Objectives and expectations of ECE programs

Zigler and Styfco (2010) report that Head Start's initial goals were based on lessons learned from the Early Training Project, an ECE program that targeted children from “extremely poor inner‐city homes headed by parents with weak education and occupational status” (p. 6), namely that (a) brief intervention cannot forever change the life of a poor child, (b) early enrichment and parental involvement can boost intellectual performance of children at risk for school failure, and (c) children's IQ gains fade over time. In fact, cognitive improvements were only a tertiary objective of the initial Head Start program, as listed in what is referred to as the Cooke Report (U.S. Department of Health, Education, and Welfare, 1965).

#### 1.3.3 Proposed theories linking ECE participation to long‐term outcomes (and impacts)

**Figure 1:**
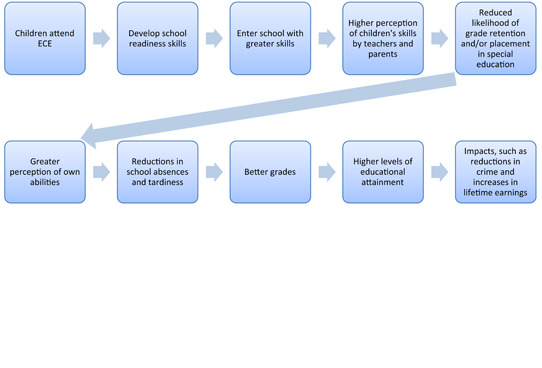
Logic model of the school competence theory

A number of theories of change have been posited to explain how ECE participation could lead to long‐term positive outcomes and impacts. Woodhead (2004) summarizes the three most common:

- *Direct effects*, wherein ECE participation “cause[s] a permanent change in children's cognitive functioning” (p. 33). In order for this theory to be validated, children's immediate cognitive gains are/should be sustained (and built upon) as they age.
- *Sleeper effects*, wherein immediate cognitive gains seem to dissipate but are, in actuality “lying dormant until some process of maturational change or environmental triggering [stimulates] their reawakening during the later school years” (p. 33) (or, alternatively, that some other change in the child goes unobserved during childhood and only appears to manifest later in life). Because this model is based on unobservable changes in the child, its presence is only deduced by the child's later achievement or situation, which makes the causal link difficult to establish.
- *School competence theory* (or, “gateway effects”), wherein initial improvements in cognitive skills, although fading over time, lead to reductions in grade retention and referral to special education in the first few years of school, resulting in more positive academic expectations and achievement (see Figure 1). According to the theory, as a result of participating in ECE and of the initial cognitive gains, children themselves gain a greater perception of their own skills and their parents and teachers will view the child as smarter and more adjusted, which in turn leads to a reduction in grade retention and referral to special education, and that leads to a more positive cycle of achievement and expectations (of parents, teachers, and the children, themselves), and that leads to greater educational attainment and other later life impacts. It is also possible, however, that the decision not to place a child in special education is a product of having worked with the child in preschool (a form of operator bias),^2^ and that the better long‐term results stem from avoiding the negative consequences or stigma of special education placements.

Cunha and Heckman (2007) posit another model (referred to as the “skills beget skills” model) that is also sometimes referenced in the ECE literature and shares some similarities with the direct effects model, wherein “the skills produced at one stage [of a child's development] augment the skills attained at later stages” (p. 35). This is an economic model of skill formation that is based on six “facts” (perhaps better described as “assumptions”) established from the authors' review of the empirical research. The model views the skill formation process in multiple stages, each corresponding to “a period in the life cycle of a child.” It relies on three principles: first, “that the skills produced at one stage augment the skills attained at later stages” (referred to as “self‐productivity”); second, that “skills produced at one stage raise the productivity of investment at subsequent stages” (referred to as “dynamic complementarity”); and third, that “together, dynamic complementarity and self‐productivity produce multiplier effects which are the mechanisms through which skills beget skills and abilities beget abilities” (Cunha & Heckman, 2007, p. 35). Magnuson and Duncan (2016), however, conclude that “the hypothesis of dynamic complementarity in early childhood currently rests on a thin empirical base” (p. 124; see also Howard‐Jones, Washbrook, & Meadows, 2012; Lubotsky & Kaestner, 2016).

Most research on the effectiveness of ECE programs does not discuss the underlying program theory (or theory of change) that links program participation to a reduction in the achievement gap. Instead, it generally looks for positive changes in various measures without consideration of the specific causal mechanism that might explain them.

### 1.4 Why it is important to do this review

Many papers and articles attempt to summarize (or, in some instances, synthesize) research on ECE programs, although there is substantial variation in the degree to which they systematically search for, screen, review, synthesize, and evaluate the extant literature. We found and reviewed more than 40 of these research reviews, of which 13 both examined ECE effectiveness and could be considered systematic reviews; that is, they (a) employ an explicit search strategy; (b) seek to identify *all* research on a subject, from *all* sources, including literature not found in peer‐reviewed journals; (c) screen studies using a set of predetermined inclusion/exclusion criteria; and (d) evaluate the quality of the studies they include and assess the validity of their findings (see Appendix A for a full list of the reviews we considered).

The others that we found either do not appear to be systematic reviews or do not investigate the effectiveness of participation in an ECE program, in which case, as we describe below, they typically compare the effectiveness of one programmatic variation to another.

Among those that we do not consider to be systematic reviews is a 2017 Brookings Institution volume produced by a blue‐ribbon panel of ECE experts that reviewed the evidence of prekindergarten (pre‐K) programs (Phillips et al., 2017).^3^ Although this volume (and its included “consensus statement”) provides much insight into the research on state pre‐K programs, it did not employ a explicit search strategy, screen studies using preestablished criteria, or apply systematic criteria based on methodological rigor to decide whether an evaluation should be included in the review. The closest it came to making an assessment of quality was to call some of the research “problematic,” without specifying which studies were and why they considered them as such.

Likewise, a similar, earlier paper by a panel comprising many of the same scholars, “reviewed rigorous evidence” (Yoshikawa et al., 2013, p. 3), providing a thoughtful summary of the evidence on preschool education programs. However, it too, does not define “rigorous” nor the methods by which studies were located and screened or how differences in study quality were taken into account. (see Appendix C for a list of reviews that did not meet the criteria to be considered systematic reviews, and which are therefore not included in our discussion of findings in Appendix B).

Most of the 40 reviews we found focus on ECE effectiveness generally, sometimes (but secondarily) drawing on programmatic differences identified during study coding to examine what program characteristics appear to be the most effective. Often, however, due to either resource limitations or other reasons, researchers narrow the focus on the review in one way or another. Some, as in the case of a systematic review by D'Onise, Lynch, Sawyer, and McDermott (2010), narrow the focus to a particular outcome domain and/or to either short‐, medium‐, or long‐term outcomes (or impacts). Others elect to limit inclusion to studies of U.S. programs (or, alternatively, non‐U.S. programs), to those of particular program types (e.g., Head Start or pre‐K), to studies published or programs operated after a certain year, or to any combination of these. For example, Duncan and Magnuson (2013) conducted a meta‐analysis of ECE programs but limited their review to immediate (measured within 6 months of program completion) cognitive outcomes and Gilliam and Zigler (2000) consider only “state‐funded preschool programs.”^4^

Other reviews (of the 40), which we did not include because they do not examine studies with a no service control or comparison group, take a comparative effectiveness approach, reviewing and synthesizing studies that compare children who receive one variation of a program to those receiving another (we expect to use these later in our analysis to show what, if any, programmatic components work better than others and what this means about the developmental plasticity of children). This approach is commonly used to identify the most effective curricula. Nguyen's (2017) meta‐analysis, for example, reviewed and synthesized 72 studies that compared preschool‐aged children that participated in programs that used targeted, domain specific curricula to those that used whole‐child curricula. Similarly, Chambers et al. (2016) included studies that compared the literacy and language outcomes of children who attended preschools that used either a “comprehensive” or a “developmental‐constructivist” approach. And Chambers et al. (2010) systematic review synthesized studies that “compared alternative approaches to ECE from 1960 to present” (p. 7), presenting average effect sizes for each of a number of curricula and programs used in preschool classrooms and grouping them by evidence of effectiveness.^5^

Others have attempted to identify whether ECE programs that include a “parenting education” component have a larger effect on children's cognitive and preacademic outcomes (e.g., Grindal et al., 2016; White, Taylor, & Moss, 1992) or what effect preschool quality has on children's outcomes (e.g., Vandell & Wolfe, 2000).

#### 1.4.1 Limitations of prior systematic reviews

Even among those 13 reviews that adopt a systematic approach, one or more weaknesses either threaten the validity of the findings or limit their usefulness (see Appendix B for a discussion of findings from these reviews).

First, those reviews with meta‐analyses typically report one average effect size for a particular domain, combining effect sizes for a wide range of outcome measures (e.g., Manning, Homel, & Smith, 2010 combined, among other things, language and math test scores, school grades, and IQ test scores into one cognitive measure) and, often, without regard for the age of the child when measured. Camilli et al. (2010) combined “intelligence and cognitive/reading achievement domains…into a single “cognitive” domain” (p. 592). Although not explicitly stated, we presume that the calculation of the average effect size of the cognitive domain involved the combination of outcomes measured immediately after program completion, throughout primary school, and even up to and including secondary school.

This raises the potential for the “apples and oranges” problem; that is, the combination of disparate outcome measures, populations (e.g., demographic composition and ages of those evaluated), and/or interventions, which threatens the validity of the review. As Sharpe (1997) argues, in meta‐analysis, it is important to strike a “balance between too broad or to narrow a focus” (p. 884). Many reviews, however, seem to paint with too broad a brush. The problem with this approach is twofold. Not only does the combination of apples and oranges potentially threaten the validity of the review, it may also obfuscate the variation in the changes across time and outcomes, thereby, limiting the usefulness of the findings. For example, one average effect size for a broad domain such as cognition, ignores that, over time, effects appear to diminish. It also ignores that there is variation in the size of the immediate effect and its general fade out. For example, McKey (1985) find modest variation in immediate effects between tests of IQ, achievement, and school readiness, but, strikingly, substantial differences in how the size of each of these outcomes decreased over time.

We think, therefore, that the better approach is to use more narrowly constructed domains, as described by the What Works Clearinghouse (see “Synthesis Procedures and Statistical Analysis,” below).

In addition, but less importantly, these reviews are not up‐to‐date and typically do not provide a full picture of ECE effectiveness. Of the 13 reviews mentioned above, only one (McCoy et al., 2017) includes studies conducted in the past 5 years, and even this one examined only medium‐ and long‐term outcomes. None of the reviews include the most recent follow‐up to the evaluation of Tennessee's pre‐K program, which found negative overall effects for ECE participation at third grade.

These reviews are also typically narrowly focused, often considering outcome measures in one domain and/or in the short‐, intermediate‐, or long‐term only. Only six reviews examined a comprehensive set of outcome measures, across a variety of domains and including those measured immediately after program completion and into adulthood. The result is that while research reviews on each of the outcome domains of interest and in the short‐, medium‐, and long‐term (including adulthood) have been completed, the findings must be collected piecemeal, with each based on analyses using different sets of studies with varying methods for identifying and screening research and for evaluating the quality of the included studies (with substantial variation in the rigor of these evaluations).

#### 1.4.2 Contribution of this review

Unfortunately, there is sharp disagreement among experts (and policy advocates) about the effectiveness of ECE programs.

ECE supporters argue that early intervention can compensate for the conditions that, at school entry, put disadvantaged children behind on various developmental dimensions—with the gap increasing as school becomes more demanding. On a selective basis, they point to various studies (e.g., the Perry Preschool Project and the Abecedarian Project) that show lasting gains in educational attainment, employment, and earnings, and substantial reductions in out‐of‐wedlock births and criminal behavior.

Naysayers argue that the ECE intervention is not strong enough to counter other forces in the child's life (such as family and neighborhood) and that these other factors are much more important to the child's cognitive and socioemotional development. They point to studies (e.g., Head Start Impact Study, Westinghouse Report) that show no or small effects or whose effects seem to disappear in a few years (“fade out”).

There is a certain selectivity in the way past studies are used to argue whether ECE programs “work,” rather than more balanced assessment of whether they can narrow the achievement gap to a socially significant degree. For example, Elango et al. (2015) claim that their review “organizes and synthesizes the literature on early childhood education” (p. 2), but includes evaluations of only 11 ECE programs (out of over 50 of which we are aware), one of which is a review of a program in Norway, that either have positive findings or, if they do not, can be easily criticized (in two of the studies, they turned nonstatistically significant findings into positive findings and increased the magnitude of already‐positive findings).

Wise policy in this area calls for as full an accounting as possible of what is known about the effectiveness of ECE programs, including how they are administered, if they are cost effective, whether one model is more effective than another, and which model works best for whom.

Setting our project apart from other studies will be its emphasis on reconciling the capacities—and needs—of parents with the ability of ECE programs and other social institutions to help provide what children need for optimal social and cognitive development. We expect the findings to recognize the reciprocal importance of parent–child (and, as appropriate, grandparent–child) relationships. Thus, we will also review the findings of other programs that seek similar outcomes, for example, programs like Parents as Teachers or Nurse–Family Partnership that seek to improve children's cognitive outcomes through a change in parental knowledge, capacity, and/or behavior.

Moreover, our review will attempt to fill a void in the ECE literature by providing an up‐to‐date synthesis of ECE's effects on a wide range of outcome measures, across cognitive, socioemotional, behavioral, and health domains (as well as crime, teen pregnancy, and economic impacts) from early childhood through adulthood. We expect to take great care to avoid combing dissimilar effects, thus reducing the “apples and oranges” threat to the validity of our findings.

Because our report will be prepared under the auspices and rigorous standards of the respected Campbell Collaboration and with the guidance of a blue‐ribbon advisory board—Jacob Klerman, Rebecca Maynard, Michael Puma, and Matthew Stagner (see Appendix D for biographical information)—we have high hopes that it will have broad policy influence.

In the current political climate, the role of the federal government in early childhood programing has become uncertain. But even if the federal government is less active in this area than previously, program expansion at the state and local level will likely continue. Although many would prefer bolder federal action, state and local action could present a greater opportunity for experimentation and innovation. In fact, many of the evaluations of the programs to be included in this review were funded and administered by organizations other than the federal government, including state and local governments, nonprofit organizations, and academic institutions.

Therefore, besides national policymakers and researchers, we will present our findings in a way that is useable by state and local planners, emphasizing the applicability of findings to particular populations, program settings, and geographical locations while also considering the applicability of decades‐old findings to present day conditions. Available evidence, for example, points to different approaches for children from severely disadvantaged households as well as for children from homes where English is the second language.

Rather than using such findings as evidence that ECE programs “work,” we hope to use them as guidelines for how programs could be structured for greater effectiveness in particular situations. In doing so, we will attempt to reconcile early learning and development objectives with child care as a means for helping parents to work in the paid economy.

In addition to reaching out to federal and state officials, we would use our long‐standing ties to the early childhood community to disseminate our findings.

## 2 OBJECTIVES

This project seeks to answer questions about the effectiveness of early childhood programs in meeting their important goals, paying careful attention to the effectiveness of program variations (especially in regard to particular demographic and economic target groups).

This review will be guided by two overarching questions:

(1) How effective are existing ECE programs when it comes to improving the cognitive, socioemotional, behavioral, and health outcomes of low‐income children? And what does the research say about these effects in the short, intermediate, and long term?
(2) What program variations are more effective than others? This inquiry comprises many more narrowly focused programmatic questions that we will attempt to answer using moderator analyses:

Do programs that provide ancillary services to parents/caregivers produce, on average, larger effects than those that provide only direct instruction to children?
What is the optimal program length (i.e., full‐year, academic year, or summer), duration (e.g., 1, 2, or more years), and intensity (i.e., half‐day or full‐day) of an ECE program?
Do low‐income students benefit more from participation in programs that primarily target and provide services to these students or from programs that are offered to students regardless of income or other disadvantage (e.g., universal state pre‐K programs)?
Does the level of teacher credential (e.g., college degree, ECE certification) influence the magnitude of effect sizes? And, if so, how?
How does the magnitude of effect sizes of U.S. programs compare to those in other countries?
How does the magnitude of effect sizes of demonstration projects (e.g., Perry Preschool, Abecedarian) compare to public programs (e.g., Head Start and state pre‐K)?
How has the magnitude of effect sizes changed since 1960?
Do programs that employ a curriculum produce larger effects sizes? And, if so, which curricula appear more effective?
Do programs that offer follow‐on services produce, on average, larger effects sizes than those that do not?
Is program focus (e.g., “whole‐child” vs. cognitive skill development) associated with differences in magnitude of effect sizes?
How does the magnitude of effect sizes change in relation to class size and/or student‐teacher ratios?

## 3 METHODOLOGY

### 3.1 Characteristics of the studies relevant to the objectives of the review

The body of evaluative literature of ECE programs is substantial and diverse, covering more than half a century and programs ranging from small demonstration projects to large‐scale public programs such as Head Start. Over that time, a wide range of research methods have been used to evaluate ECE programs, including randomized controlled trials, matched comparison group studies, and, more recently, regression discontinuity designs (RDDs) and econometric analyses using large, national datasets.

Typically, these research studies attempt to estimate the effect of ECE participation on one or more child development domains, which are cognitive (including achievement, intelligence, and school progress), socioemotional, behavioral, and health outcomes, as well as later outcomes such as crime, teen pregnancy, earnings, and use of or reliance on welfare assistance. Some studies provide longitudinal assessment of these outcomes, tracking the same children beginning at program enrollment, through elementary school, and up to and including adulthood. Others measure immediate effects only or use survey data to estimate long‐term effects (or, impacts) without attempting to measure earlier outcomes.

The following three evaluations are prominent works, often cited in the discussion of whether ECE “works,” that we expect to be representative of the studies included in our review and the variation of the ECE research base. In addition, we identify some of the methodological concerns for each.

- *The High/Scope Perry Preschool Project* (hereafter, “Perry Preschool Project”), which operated in Ypsilanti, MI between 1962 and 1967, sought to help “children who were at high risk of school failure to successfully develop the intellectual abilities needed to succeed in school and thus graduate from high school” (Schweinhart, Barnes, & Weikart, 1993, p. 5) by providing a combination of a part‐time preschool program and home visits to 58 3‐ and 4‐year‐olds (65 children were assigned to the control group). The evaluation of the program, led by Lawrence Schweinhart and David Weikart, was a pioneering effort to evaluate rigorously, using a combination of random assignment and matching, the long‐term impact of an ECE program. Not only did the project's creators implement a program highly regarded by developmental experts, but they also thought ahead to do so in a way that would be conducive to later experimentation, tracking the study participants' cognitive, socioemotional, and behavioral development, and, eventually adult impacts from the 1960s into the late‐1990s and early 2000s (most recently through age 40). Despite the obvious care the research team devoted to the evaluation, several major issues related to the random assignment process and data inconsistencies limit the confidence that can be placed in these findings.
- *The Head Start Impact Study* was a federally mandated national assessment of the federally funded Head Start program, conducted by Westat, the Urban Institute, the American Institutes for Research, and Decision Information Resources. Begun in 2002, this was the first national study of Head Start to use random assignment to evaluate its effectiveness, randomly assigning a sample of nearly 5,000 Head Start eligible children from 23 states to either a treatment or a control group. Data on children's cognitive, socioemotional, behavioral, and health outcomes were collected through 2013 when the children completed third grade. Although children were randomized to a treatment condition, a frequent criticism of this study is that control group children were able to (and to a substantial degree did) participate in other ECE programs, including other Head Start centers, which poses the potential for both substitution and crossover bias.
- *Oklahoma's pre‐K program*, a publicly funded universal (meaning, open to all age‐appropriate children, regardless of family income or other disadvantage) preschool program, was established in 1998 and currently serves about 70% of all eligible 4‐year‐olds. In 2003, William Gormley, Ted Gayer, Deborah Phillips, and Brittany Dawson used a regression‐discontinuity design and a sample of more than 2,800 children to compare the test scores of children in the Tulsa Public School District who had attended pre‐K with those who were about to enter pre‐K, reporting separately findings for all racial and ethnic groups (Gormley & Gayer, 2005). Due to the research design used (those in the comparison group eventually completed pre‐K), the children used in the sample were not followed as they aged and progressed through school, and thus it is unclear if the initial positive effects were maintained or faded over time. Moreover, the reported effect sizes are for children with birthdays within 12 months of the cut‐off which are somewhat higher than the effect sizes for children with birthdays within 3 or 6 months of the cut‐off, which calls into question the comparability of the two groups. In addition, as described below, there are concerns about using age as a cut‐off variable and its effect on the validity of the estimates obtained.

### 3.2 Criteria for including and excluding studies

#### 3.2.1 Types of interventions

Eligible studies should evaluate center‐based, classroom‐style programs, defined as programs that take place in classroom‐like settings in public school classrooms, faith‐based institutions, or other public or private centers and that provide educational instruction directly to children, operating regularly for at least one academic year (approximately 180 days). Programs will not be excluded if they also provide ancillary services (such as parental or medical services).

Eligible programs need not provide curriculum‐based instruction, be staffed by certified teachers, nor operate for a particular minimum number of hours per day. We will, however, collect information on these program characteristics during coding and, provided we have enough studies, use them as moderators in our analysis. We expect to use these moderators to learn about the plasticity of children's learning and what program variations might improve their skills and achievement.

Studies that use student outcomes to measure or compare the effectiveness of teachers or curriculum will not be included in our meta‐analysis. These studies, however, might contain evidence that is relevant to our review, and we will, therefore, make note of these studies so that we might use them to interpret our findings.

#### 3.2.2 Types of participants

Eligible studies should investigate the effect of an applicable intervention that serves disadvantaged children from low‐income families sometime from birth to age five (with age breaks). We define “disadvantaged” or “low income” as below twice the official U.S. poverty line (or threshold, as adjusted for family size). Our preliminary research, however, found that many studies use these terms without providing a definition, or that the programs targeted children using factors other than income.^6^

We have found that the following terms are compatible and used frequently in the literature: “eligible for free or reduced price lunch” (or “FRPL”), “low income,” “low socioeconomic status,” “poor,” and “at risk.” Studies that use these terms (or slight variations) to define the participants will be eligible for inclusion. We will base this decision on a combination of both the study author's description of the program as well as information reported about the characteristics of the enrolled students. We will capture information about the composition of the sample during study coding and use this information in moderator analyses.

When studies evaluate universal programs that serve children from all SES backgrounds, we will only include them if effects for low‐income children are reported separately. When reported, however, these effects are usually the result of subgroup analyses, which are subject to issues related to sample size and statistical power. We will not, therefore, combine the results from universal programs and targeted programs but, instead, will report their results separately. We will also collect information on the percent of enrollees in these universal programs that are from disadvantaged families and, perhaps, use this as a moderator in our analysis.

We will exclude studies that target children with severe developmental disabilities, learning disabilities, or chronic medical disorders.

#### 3.2.3 Types of outcome measures

Eligible studies should either report at least one immediate (or “short‐term”) outcome measure or be a follow‐on to a study that reported one (a “follow‐on” is defined as a study that reports intermediate outcome or impact measures of a program on a group or groups that have previously been evaluated).^7^

In addition, the outcome measure(s) reported should fall under one (or more) of three broad categories:

- *Cognitive*, such as school performance; language, reading, and math skills; grades; and school performance;
- *Health*, such as health status, height and weight (including body mass index, BMI), obesity, and receipt of dental care; and
- *Socioemotional*, such as activity level, aggression, attention, activity level, internalizing and externalizing behaviors, organization/impulse control, sociability, and suspension/expulsion;

More precise outcome domains are described under “synthesis procedures and statistical analysis” below.

#### 3.2.4 Types of study designs

Eligible studies should investigate the effectiveness of participation in ECE programs compared to nonparticipation (the counterfactual) and report primary research findings (as opposed to being cross‐study analyses or commentary) with at least one eligible outcome measure.

We do not intend to use study design alone as a criterion to make inclusion/exclusion decisions; rather, our plan is to rigorously review each eligible study for threats to causal validity, as described below (see Section 3.5), and exclude those that are rated as *Insufficient Causal Validity*. We think this is the best approach because it should provide a more precise justification for the decision to include or exclude each study.

In order to be included, a study must employ either a comparison group, provide a comparison time series, or use a control variable that is related to the extraneous variable.^8^

We will include three‐pronged studies that investigate the effectiveness of supplemental instruction or tutoring for children already in preschool classrooms only if one of the group's does not receive any preschool services. Without a no‐preschool group, these studies are comparative effectiveness evaluations and, as such, do not measure the effectiveness of participating in ECE. As with studies that use student outcomes to measure the effectiveness of teachers or curriculum, we will use these studies to interpret our findings and identify what, if any, programmatic variations work better than others and what this means about the developmental plasticity of children.

#### 3.2.5 Other criteria

Eligible studies should have appeared after 1960 and been written (or be available) in English. In addition, these studies should have been conducted in the United States or, because of their social and economic conditions, and research and evaluation infrastructures, a Western European country, as defined by the World Bank (i.e., Austria, Belgium, Denmark, Finland, France, Germany, Greece, Iceland, Italy, Luxembourg, Norway, Portugal, Spain, Sweden, Switzerland, the Netherlands, and the UK), or one of the following English‐speaking countries: Australia, Canada, or New Zealand.^9^

#### 3.2.6 Application of the criteria

We expect to apply our inclusion criteria in two stages. In the first, the title and abstract for each reference will be screened using the following set of general questions:

1. Is the study reported or available in English?
2. Was the study published after 1960?
3. Does the study report primary research findings (as opposed to being a cross‐study analysis or commentary)?
4. Is the study an outcome or impact evaluation and does the study report at least one cognitive, socioemotional, behavioral, or health outcome or impactfor children?
5. Does the study attempt to attribute these outcomes or impacts to children's participation in what appears to be a preschool‐type program?
6. Did the program provide educational instruction directly to children?
7. Were children selected for participation because of a severe developmental disability, learning disability, developmental delay, or chronic medical disorder?
8. Is there another apparent reason (i.e., wrong age group or nonapplicable country) for excluding this study?
9. Is the study eligible for inclusion at this stage?

Given the large number of expected results, we will have two reviewers independently double screen the first 15% of references in order to establish inter‐rater reliability. Reliability will be assessed by calculating Cohen's *κ* for the reviewers' responses to question eight. If inter‐rater reliability meets or exceeds 0.80, the remaining references will be split in half with one half assigned to each reviewer. If *κ* falls below 0.80, discrepancies will be resolved by a third reviewer. In this case, another sample of references will be double screened and inter‐rater reliability reassessed (this iterative process will continue until satisfactory inter‐rater reliability is achieved).

Alternatively, if a *κ* of 0.80 seems impractical after a number of iterations, we would double screen all remaining studies and exclude only those for which both reviewers agree are not relevant.

In order to screen studies efficiently, we will immediately exclude a study when it fails to meet any one criterion, even if the exclusion happens after the first question of the screening tool. Studies that do not meet the criteria in stage one will be excluded from the review. In the event that there is insufficient information to answer one (or more) of the screening questions, the question will be marked as “cannot tell” and the study will remain eligible for the next stage of screening (the study may be excluded later, however, if the missing information is found during the full‐text screening and it no longer meets the criteria in stage one).

The studies that remain eligible after stage one will be subjected to a full text screening using the following set of more focused questions:

1. Was the study conducted in the United States or one of the following countries?
  a. Australia
  b. Austria
  c. Belgium
  d. Canada
  e. Denmark
  f. Finland
  g. France
  h. Germany (or West Germany, pre‐1990)
  i. Greece
  j. Iceland
  k. Italy
  l. Luxembourg
  m. New Zealand
  n. Norway
  o. Portugal
  p. Spain
  q. Sweden
  r. Switzerland
  s. The Netherlands
  t. UK

1. Did the intervention target children sometime after birth to age five? (Select “yes” for studies evaluating Head Start, Early Head Start, or state pre‐K.)
2. Are participants described as, or are effects reported separately for children described as, the following? (select “yes” for studies evaluating Head Start or Early Head Start).
  a. At‐risk
  b. Disadvantaged
  c. Low‐income
  d. Low socioeconomic status
  e. Poor
  f. FRPL eligible
3. Did the program provide educational instruction to children on topics such as math, language, and science as a primary component of the intervention? (select “yes” for studies evaluating Head Start, Early Head Start, or state pre‐K).
4. Did the program provide only supplemental instruction or tutorial services to children?
5. Does the study use student outcomes to evaluate the effectiveness of teacher credentials (or other characteristics), professional development programs for teachers, pedagogical approaches, or curriculum? (these are often compared to business‐as‐usual or other variations/alternatives).
6. Did the intervention occur primarily in a center (public or private), faith‐based setting (church or other religious institution), or public school? (select “yes” for studies evaluating Head Start, Early Head Start, or state pre‐K).
7. Did the program operate for at least one academic year (approximately 180 days)? (select “yes” for studies evaluating Head Start, Early Head Start conducted after the Fall of 1965, or state pre‐K).
8. Does the study report at least one immediate outcome or is it a follow‐on to one that did so?
9. Does the study employ either a comparison (or control) group, comparison time series, or control variable?
10. Is the study eligible for inclusion at this stage?

As with the first stage, the first 15% of the references will be independently double screened and Cohen's *κ* will be calculated to measure inter‐rater reliability using the responses to question ten. If *κ* is satisfactory (0.8 or greater), the remaining references will be equally distributed among the reviewers. If not, a third reviewer will resolve the discrepancies and the process will repeat until satisfactory reliability is achieved. Or, if the desired *κ* does not seem attainable, we will double screen the remaining studies and only exclude studies that both reviewers agree should be removed.

In the event that there is insufficient information with which to answer one (or more) of the screening questions, the answer will be marked as “cannot tell” and the study will remain eligible for inclusion. Studies that meet the criteria in both stages will be assessed for risk of bias (see below).

If it is later determined (during the risk of bias assessment or full coding) that a study was improperly screened, it may be excluded from the analysis, and an explanation will be provided in the review.

### 3.3 Search strategy

We will attempt to find as many English‐language studies of ECE programs as possible, including gray literature. To do so, we expect to search numerous electronic databases, the websites of research and advocacy organizations and government agencies, and a number of international trial registers, as suggested by the Campbell Collaboration. In addition, we will conduct hand searches of high‐priority journals and review the references of published systematic reviews and meta‐analyses.

We will use Zotero to manage our references and, when all searches are complete, to remove duplicates.

#### 3.3.1 Database searches

The bulk of our electronic database searches will be conducted using EBSCO*host*, a collection of approximately one hundred indices and databases. We will individually search each of the following databases using the EBSCO*host* interface (this is the only interface available to us through the University of Maryland libraries with which to search these databases):

- Academic Search Complete
- Academic Search Premier
- Chicano Database
- Criminal Justice Abstracts
- eBook Collection
- EBSCO eClassics Collection
- EconLit
- Education Index Retrospective: 1929–1983
- Education Source
- Education Resource Information Center (ERIC)
- Family and Society Studies Worldwide
- Family Studies Abstracts
- Historical Abstracts with Full Text
- National Criminal Justice Reference Service Abstracts
- Open Dissertations
- Primary Search
- Professional Development Collection
- PsycARTICLES
- Psychology and Behavioral Sciences Collection
- PsychINFO
- Race Relations Abstracts
- Social Work Abstracts
- SocINDEX with Full Text
- Teacher Reference Center
- The Nation Archive
- The National Review Archive
- The New Republic Archive
- Urban Studies Abstract

Entries in these databases and indices are well catalogued and include reference information and an abstract for each. Thus, we have designed the following search phrase, which concatenates keywords using the boolean operators “OR,” and “AND” to search only the abstracts for each entry:

(preschool OR pre‐school OR prekindergarten OR pre‐kindergarten OR pre‐K OR preK OR “Head Start” OR “early childhood education” OR “early childhood care and education” OR “early N5 education” OR “nursery school” OR ECCE OR ECE) AND (experiment* OR quasi‐experiment* OR quazi‐experiment* OR “random* control* trial” OR “random* assignment” OR RCT OR trial* OR “control* study” OR “control* studies” OR “control* design*” OR “control* trial*” OR “control* group*” OR “control group design” OR “trial registration” OR “random* allocat*” OR evaluation OR “comparison group” OR outcome* OR impact* OR effect*) AND (achievement OR behavior* OR behaviour* OR cognit* OR intelligence OR IQ OR knowledge OR “school readiness” OR socioemotional OR socio‐emotional OR socialemotional OR math* OR literacy OR vocabulary OR “language skills”) AND (child*) AND (poor OR “low income” OR “low‐income” OR disadvantage* OR “at risk” OR FRPL OR “eligible for free school meal* OR universal* OR “state pre‐K”)

We will not use date or language of publication as search limiters; rather, we will screen studies for date and language relevance during stage one of our inclusion criteria.

In addition to our EBSCO*host* search, we plan to search individually the following databases:

- JSTOR
- OpenGrey
- Web of Science Core Collection: Citation Indexes
- WorldCat

For Web of Science, we will use a similar procedure to which we will use for EBSCO*host*, except that we will apply the search phrase to the abstracts, titles, and keywords (referred collectively to as “topics” in Web of Science) of the references they contain instead of the abstracts only.

The search tools of the other three, however, are either limited to shorter search phrases or do not allow for (or, in some cases, discourage) the search term to be applied only to abstracts, and thus we will use the following, shorter boolean search phrase when searching them:

(preschool OR prekindergarten OR “nursery school”) AND (poor OR “low income” OR disadvantage*) AND (effect* OR outcome* OR impact*) AND (achievement OR cognitive OR health OR soci?emotional) AND (experiment OR quasi‐experiment OR quazi‐experiment OR random*) NOT (autis*)

If a search returns no results, we will search again using only the keywords contained in the first set of parentheses in the longer search term.

#### 3.3.2 Other online searches

Our search will include reviewing the websites of research firms, advocacy groups, institutions of higher learning, and government agencies for technical reports, white papers, and conference proceedings. We will search the following websites:

- Abt Associates
- Administration for Children and Families' National Research Conference on Early Childhood
- American Educational Research Association
- American Institutes for Research
- Center for Education Policy
- Center for Research and Reform in Education
- Center for Research in Educational Policy
- Evidence for Policy and Practice Information and Coordinating Centre (EPPI‐Centre)
- Chapin Hall
- Danish Clearinghouse for Educational Research
- Education Endowment Foundation
- European Early Childhood Education Research Association
- Frank Porter Graham Child Development Institute
- Mathematica Policy Research
- MDRC
- National Center for Education Research
- National Education Association
- National Association for the Education of Young Children
- National Institute for Early Education Research
- NBER Working paper Series
- OECD
- Peabody Research Institute
- RAND Corporation
- Regional Educational Laboratories
- U.S. Department of Health and Human Services
- Westat
- What Works Clearinghouse
- Worldbank

We will also search by hand the conference proceedings from the following United States and international conferences to identify those not yet indexed in the commercial databases.

- Annual International Conference on Childhood Education (https://10times.com/childhood-education)
- Early Childhood Care and Education International Conference https://www.openingmindsusa.org/)
- Early Years Conference (https://interprofessional.ubc.ca/initiatives/earlyyears2020/)
- National Association for the Education of Young Children (NAEYC) Annual Conference (https://www.naeyc.org/events/annual)
- National Head Start Conference (https://www.nhsa.org/event/2020-national-head-start-conference)
- National Research Conference on Early Childhood (http://nrcec.net/)
- Opening Minds Early Childhood Education Conference (https://en.ecceconference.com/)

We will also use Google Scholar to search for additional studies, using various combinations of the keywords in our search phrase (search terms cannot exceed 32 words when using Google Scholar, and thus we cannot use our longer search phrase). Our preliminary searches yielded more than 100,000 results, which is far more than we can realistically screen, and thus we will limit the number of results to those contained in the first 20 pages (this appears to be a common approach in other systematic reviews, including Campbell Collaboration reviews).

#### 3.3.3 Handsearches

We will conduct handsearches of 10–15 high‐priority journals. The journals to be searched will be identified using the results from our database searches and based on their availability at the University of Maryland's libraries. Those journals available at the library and containing the greatest number of references returned in our results will be considered high‐priority.

In conducting our handsearches, we will review the table of contents of each volume since 1960, record in Zotero any studies that match our search criteria, and apply our inclusion criteria on‐the‐spot (rather than adding to our reference database and applying at a later date).

#### 3.3.4 Previous systematic reviews

During the preparation of our title registration and the description of prior reviews (see Appendixes A through C), as well as subsequent grant writing activities, we established a list of previous systematic reviews, meta‐analyses, and literature reviews conducted in the area of ECE. We will use the references of these reviews to identify any studies that may have been missed during the first two stages of the search. Any studies that may be related to our topic will be included in our reference list for future screening.

#### 3.3.5 Trial registers

The Campbell Collaboration provides a list of 22 trial registers. We will review these registers for any ongoing, concluding, or future evaluations of ECE programs. While these studies may not be completed in time to be included in this review, they may be useful in future updates.

### 3.4 Data extraction and study coding procedures

Studies that have met our inclusion criteria and either *Meets Standards without Reservations* or *Meets Standards with Reservations* (see “Risk of Bias” below) will undergo full coding. Two reviewers will apply the coding instrument to these studies (a copy of the coding instrument can be found in Appendix E). The coding instrument is designed to extract the following information:

- Study characteristics (e.g., authors and affiliation, year of publication, country of origin, and study design);
- Intervention characteristics (e.g., the year[s] the intervention began/ended, children per class or child‐staff ratio, dosage and duration of the intervention, intended vs. actual dosage, characteristics of teachers and classrooms, services provided, and any services comparison group children may have received);
- Participant characteristics (e.g., age, race, gender, language status, characteristics of mother, and parent marital status); and
- Outcome measures (e.g., domain of outcome and descriptive statistics).

When coding for outcome measures, we will attempt to ascertain whether the effects reported are intent‐to‐treat or treatment‐on‐the‐treated estimates and if the measure has face validity.

We anticipate that we will have too many studies to allow for double coding of each study, and therefore we expect to establish a sample of studies (approximately 15%) that will be independently double coded. Where possible, we will use the appropriate test (e.g., Pearson correlation, Cohen's *κ*, intraclass correlation) to measure inter‐rater reliability. If inter‐rater reliability is not satisfactory (e.g., *κ* < 0.80) the reviewers will meet and resolve discrepancies by consensus. Another sample of studies will be independently double coded and inter‐rater reliability will be reassessed. When inter‐rater reliability is deemed satisfactory, the remaining studies will be divided in half and each reviewer will be assigned one.

### 3.5 Risk of bias

Our plan to assess study quality and address possible issues with the causal validity of the included studies involves both a formal study rating process that occurs before final inclusion decisions are made and studies are fully coded as well as the use of a number of moderators in the synthesis and analysis stage of the review.

#### 3.5.1 Study rating process

Those studies that meet our criteria for inclusion will be subjected to an assessment of their causal validity prior to full study coding and, based on this review, will be assigned an overall rating of either *Strong Causal Validity*, *Sufficient Causal Validity*, or *Insufficient Causal Validity*.^10^

To make these decisions, we have created a causal validity assessment tool. This tool is a modified version of the What Works Clearinghouse's (WWC) rating standards, and it draws from a number of additional sources, that is, the *Cochrane Handbook for Systematic Reviews of Interventions (Version 5.1.0)* (Higgins & Green, 2011), *The Handbook of Research Synthesis and Meta‐Analysis* (Cooper, Hedges, & Valentine, 2009), *The Maryland Scientific Methods Scale* (Farrington, Gottfredson, Sherman, & Welsh, 2002; What Works Centre for Local Economic Growth, 2016), as well as our own work (Besharov, 2016).

Our rating tool comprises two standards, each composed of two or more criteria, meant to examine broadly the degree of causal validity in each study. These standards evaluate the extent to which the methods used by the researchers appear to account for threats to causal validity that may be the result of selection bias or attrition (as defined below).^11^ This tool is tailored to be applicable across a wide variety of study designs, including comparison‐to‐self designs.

As described below, each standard will be rated according to the extent to which the study satisfies the set of criteria that comprise it. Accordingly, the standards will be rated (borrowing language from the WWC) as either *Meets Standard without Reservations* or *Does Not Meet Standard*, with the exception of the selection standard, which can also be rated as *Meets Standard with Reservations*.

When each standard has been rated, an overall study rating will be assigned as follows:

*Strong Causal Validity*, if the study meets each of the standards without reservations. The only studies eligible for this rating, therefore, would be properly conducted randomized control trials (RCTs; likewise, only RCTs are eligible for the WWC's highest rating). Studies that evaluate and/or compare multiple variations of a specific program (e.g., comparing full‐day to half‐day programming) would be eligible for this rating, provided that the participants were randomly assigned and that there is a nonprogram group that did not receive services from the program.
*Sufficient Causal Validity*, if the study satisfies the selection standard (but does not use random assignment or if randomization was not performed correctly) and it meets the attrition standards (comparison‐to‐self designs are exempt from meeting the selection standard, see below).
*Insufficient Causal Validity*, if the study fails to meet any of the standards.

One caveat to these rating rules is in the case of studies that use an age cutoff RDD. These studies typically use strict age cutoffs to assign children to either a program or nonprogram group. When limiting the comparison to children just above and just below the cutoff, the assignment is treated “as if” randomization has occurred. These studies, however, may have too few children near the cutoff to generate precise estimates, and thus the researchers may include children further from it to compensate. Presumably, the further children are from the cutoff, the more dissimilar they are, which threatens the causal validity of the findings. Indeed, Thistlewaite and Campbell (1960) argue that as observations further from the cutoff are added, the inferences from RDDs become “more and more suspect.” Moreover, these results may not be directly “directly comparable to either intent‐to‐treat or treatment‐on‐the‐treated estimates from experimental studies” (Duncan & Magnuson, 2013, p. 119; see also, Gibbs, Ludwig, & Miller, 2011; Lipsey, Weiland, Yoshikawa, Wilson, & Hofer, 2015).

Given these two limitations, we will include age cutoff RDDs, but consider them separately, provided they meet the attrition standard of the *Sufficient Causal Validity* rating as outlined above.

We expect to use these overall study ratings to reduce the risk of bias in our analysis in two ways. First, we will exclude any studies that are rated as *Insufficient Causal Validity*, because, if included in our analysis, their findings, perhaps attributable to one or more of the threats to causal validity, might bias our results. Second, with the remaining studies, we will use the study rating as a moderator in our analysis. Provided we have enough studies, we plan to conduct sensitivity analyses in order to decide whether the results from studies with *Strong Causal Validity* should be combined with those of *Sufficient Causal Validity*. We anticipate that the results from these analyses will suggest that there is a statistically significant correlation between study rating and effect size and that we would therefore conclude that these two groups should be analyzed separately.

##### Standard 1: Selection

The selection standard only applies to studies that use a comparison group, and it comprises two criteria:

*Assignment to condition*. This criterion evaluates whether participants were randomly assigned to either a program or a nonprogram group, and it is only satisfied when the assignment was, in fact, random. A study does not meet this criterion if a combination of random assignment and matching was used, if randomization was improperly conducted (e.g., the researchers assigned children by last name, birth date, or social security number), or if, after randomization, the resulting groups were dissimilar on baseline characteristics and, therefore, required a statistical adjustment (see below).
*Baseline equivalence*. This criterion evaluates the extent to which the groups being compared are similar as measured using baseline measures, including pretest scores and demographic characteristics. It is satisfied if the study meets the baseline equivalence requirement set forth by the WWC. Accordingly, we will calculate an effect size to measure the variation between the groups on pretest scores and for each baseline characteristic reported. We will consider groups to be sufficiently similar when the effect sizes are ≤0.05*SD*. When an effect size is between 0.05*SD* and 0.25*SD*, we will require that the study authors have conducted and reported a statistical adjustment (either a regression adjustment or analysis of covariance). Effect sizes >0.25*SD* will be considered unequal, regardless of any adjustments made, and will, therefore, not satisfy the baseline equivalence criterion.

We will, however, consider the following methods as sufficient to obviate the baseline equivalence criterion: propensity score matching, fixed effects, and difference‐in‐differences.

The selection standard will be rated as follows:

*Meets Standard without Reservations*, if both criteria are satisfied;
*Meets Standard with Reservations*, if the selection to condition criterion is not met but the baseline equivalence criterion is satisfied; or
*Does Not Meet Standard*, if the baseline equivalence criterion is not satisfied.

##### Standard 2: Attrition

The attrition standard comprises two criteria:

*Reporting of attrition information*. This criterion evaluates whether attrition information is reported for all groups in the study, and thus it is satisfied only when this information is included in the study.
*Study attrition*. This criterion evaluates the degree of attrition in each study, and it is satisfied if the level of attrition does not exceed the WWC's attrition guidelines for ECE interventions (U.S. Department of Education, 2014a). These guidelines are model‐based and empirically supported, providing two acceptable boundaries (“conservative” or “liberal”) for overall and differential attrition that the WWC estimates will yield attrition bias <0.05*SD*—a level the WWC deems to be sufficiently small (Deke & Chian, 2017; U.S. Department of Education, 2014b). Unlike the WWC, we believe that the causes of attrition in ECE evaluations are often endogenous to the program, especially the quality of the program. Thus, we expect to use the conservative boundary when considering overall and differential attrition.

The attrition standard will be rated as follows:

*Meets Standard without Reservations*, if both criteria are satisfied; or
*Does Not Meet Standard*, if either criterion is not satisfied.

#### 3.5.2 Moderators

As mentioned above, we consider contamination and implementation weaknesses to also be threats to causal validity, but we have decided to use them as moderators in our analysis instead of as standards or criteria in our study rating process. We think that this approach is best because, based on our review of a number of systematic reviews, these threats are not commonly assessed during the quality appraisal process in other systematic reviews. Moreover, we are worried that the effect of using them as standards or criteria might be to systematically exclude studies that have downward biases in their impact estimates. We think that it would be valuable to know if there is evidence that, despite the likelihood of downward biases in the impact estimates, a program found some evidence of effect. Our plan, therefore, is to treat contamination and implementation weaknesses as moderators, rating each threat during coding as either low, medium, or high, as described below. We plan to review the effects of this approach during the analysis stage.

For contamination, we will rate the threat overall as well as examine and rate four distinct types: substitution bias, crossover, diffusion of treatment, and contagion (this applies only to studies that use a nonprogram group).

##### Contamination

This moderator evaluates, overall, the extent to which members of the nonprogram group receive the intervention (“crossover bias”), something similar (“substitution bias”), and are affected by it (“diffusion of treatment” and “contagion”) (see below for how each of these types of contamination will be rated). We will rate this threat as follows: low, if the threat is low for each of the four types of contamination; medium, if the threat is medium for only one or two of the types and is low for the others; and high, if the threat is high for any one of the four types or if the threat is medium for more than two.

*Substitution bias*. This moderator evaluates the extent to which the nonprogram group receives services that are similar to those received by the program group (but not from the program).^12^ We will rate the threat of substitution bias as follows: low, if the rate of substitution is <25%; medium, 25%–75%; and high, more than 75%.
*Crossover bias*. This moderator evaluates the extent to which those assigned to the nonprogram group end up in the program group and receive its services (or vice versa). Using a rating system similar to the one employed by Duncan and Magnuson (2013), we will rate the threat of crossover bias as follows: low, if the rate of crossover is <1%; medium, 1%–10%; and high, more than 10%.
*Diffusion of treatment*. This moderator evaluates the extent to which those outside the program group are directly exposed to the intervention, and potentially affected by it. To make a rating decision, we will review each study for any discussion of diffusion of treatment and consider the program's design and adequacy of the study design in limiting this form of contamination. We will rate the threat of diffusion of treatment as follows: low, if there is no evidence that children outside of the program were directly exposed to any component of the intervention; medium, if the direct exposure to the program was limited to ancillary services only; and high, if children outside of the program group were directly exposed to the classroom‐based instruction component of the program.
*Contagion*. This moderator evaluates the extent to which those outside the program group are indirectly exposed to the intervention through their interaction with members of the program group who have changed in some way and, in turn, change the nonprogram group. As in the case of diffusion of treatment, we will make a rating decision by reviewing the study and considering whether the study design increases or decreases this threat. We will rate the threat of contagion as follows: low, if there is no evidence of interaction between the groups in the study; medium, if there is evidence of an interaction between the groups but the groups are of different ages and the effect of this interaction is not likely to be significant (as perceived by the reviewer); and high, if there is evidence of interaction between the groups and the groups are of similar aged children and/or if the likelihood that the interaction had a significant effect on the nonprogram children is high.

##### Implementation weaknesses

This moderator evaluates both the quality of the program and the degree to which the program was implemented with fidelity. Regrettably, we do not expect to be able to rigorously assess implementation for each program, so if the study authors do not comment on implementation then we will answer the question as “cannot tell.” Otherwise we will rely on study authors to comment on whether the program was implemented with fidelity. We realize that this is not optimal, but we think that it deserves consideration when a study reports an implementation issue.

##### Reporting bias

Like Wilson et al. (2016), we will primarily address potential reporting bias in two ways: First, in order to reduce the threat of publication bias, we will search for and include relevant unpublished work including dissertations, technical reports, and working papers. If possible, we will use publication status as a moderator in our analysis to identify any apparent effect it may have in the variation of effect sizes. We will only conduct a formal statistical analysis of publication bias (e.g., funnel plot, regression methods, and trim and fill methods) if sufficient data permit.

Second, to reduce the threat of outcome reporting bias, in the event that we cannot calculate an effect size for a reported outcome, we will discuss the finding, especially as it relates to our results, and provide a list of any such outcomes in a table. According to Wilson et al. (2016), these outcomes may be more likely to have negative or null effects, and thus our results may be biased downward by not including them in our analysis.

In addition, to reduce the risk of location bias, we will search for and include as many relevant English‐language studies as possible, conducted, as mentioned above in the United States, Western Europe, Australia, Canada, and New Zealand.

We realize that these approaches do not account for potential biases related to the language of publication or time to publication, and they only partially account for possible bias related to publication status, selective reporting of outcomes, and location of the study. We believe, however, that the threat from these potential biases is small and our approach is sufficient to balance the need for addressing these minor threats and the limited resources available to do so.

### 3.6 Statistical procedures and statistical analysis

#### 3.6.1 Software

We will use R to calculate effect sizes (and their 95% confidence intervals) for the outcomes reported in the study findings, to combine effect sizes, and to perform moderator analyses.

#### 3.6.2 Effect sizes

The majority of ECE studies report some measure of academic achievement (or other outcome) measured as a continuous variable, including reading and/or math test scores. For these, we expect to use the standardized mean difference as our index of effect, and although both Cohen's *d* and Hedges' *g* estimates tend to be biased upwards, the latter provides a more precise measure of effect when dealing with small sample sizes and when the *SD*s of the groups in the study are dissimilar (Ellis, 2009). Since many of the early studies of Head Start (e.g., Krider & Petsche, 1967; Larson & Olson, 1968; Nummedal & Stern, 1971) had small overall sample sizes and/or had treatment and comparison groups that were dissimilar in size (and are therefore likely to have dissimilar *SD*s), Hedges' *g* should produce a more conservative and accurate estimate under these conditions, and thus we plan to use it as our primary index of effect. This should produce a more conservative and more accurate estimate under these conditions. Furthermore, this is a common approach in meta‐analyses of ECE programs, and the preferred option in WWC reviews (U.S. Department of Education, 2014a).

To calculate Hedges' *g*, we will use the formula proposed by Hedges (1981), and therefore, in order to be included in the meta‐analysis, studies must report means, sample *SD*s, and sample sizes for each outcome measure and for each group in the study.

When studies report outcome measures that are dichotomus (e.g., placement in special education), we will use the odds ratio as our measure of effect. Occasionally, studies may vary in how they measure certain outcomes, sometimes measuring a given outcome construct as dichotomous and sometimes as continuous. In these situations, we will convert the dichotomous effect size first to Cohen's *d* and then to Hedges' *g,* as described in Borenstein et al. (2009).

#### 3.6.3 Missing data and other issues

Although we expect missing data to be an issue in only a small percentage of studies, when the necessary information (i.e, means, *SD*s, and sample sizes) is not included in a study, we will not impute missing values (except for sample sizes when sufficient information is reported), but, instead, contact the study authors to request the missing data. If we are unable to obtain the necessary statistic, we will not include the result in our analysis. We would, however, discuss the finding in our report, especially as it compares to our results. In the event that an outcome is reported as an effect size in another form (e.g., *r* or Cohen's *d*), we will perform the necessary transformation using the appropriate conversion formula as described in Borenstein et al. (2009) (chapter seven of Borenstein et al., 2009 details how to perform transformations of effect sizes, converting either correlational or binary data to Cohen's *d* and from Cohen's *d* to Hedges' *g*).

In our initial searches, we did not find any evaluations using prepost, comparison‐to‐self designs. Should one or more study using this design be included, however, we would calculate an effect size using Glass' Δ—the typical practice whereby the mean of the outcome measure posintervention is subtracted from the mean of the measure preintervention and is then divided by the *SD* of the preintervention measure (Lipsey & Wilson, 2001). We will not combine these effect sizes with those calculated using Hedges' formula (or with odds ratios) because the effect sizes are dissimilar. Moreover, effect sizes from comparison‐to‐self designs tend to be significantly greater than for studies using a comparison group (which may be the result of autocorrelation), and thus the effect sizes from these different methodologies should not be combined even if the statistics were similar (Beretvas & Chung, 2008; Durlak, 2009).

If the comparison‐to‐self design does not include both a pre‐ and postintervention measure (e.g., postonly designs), we will include the findings in our review, but not in the meta‐analysis, and they would be mentioned in our discussion of results as either supporting or contrasting evidence.

We are not aware of any evaluations of ECE that use a cluster‐randomized design; however, should one be included in our analysis, we would calculate its effect size using the WWC's guidelines for doing so. In addition, we would apply the clustering correction described by the WWC to adjust the standard error and *p*‐value for each effect size.

#### 3.6.4 Outliers

Before data are combined, we will arrange each of the effect sizes and their confidence intervals by outcome domain (see below), and visually inspect them for apparent outliers. For each identified outlier, we will calculate the sample‐adjusted meta‐analytic deviancy (SAMD) statistic developed and described by Huffcut and Arthur (1995) to measure the degree to which the outlier effect size varies from what should be expected by chance. Given that the SAMD approximates a *t* distribution, any result of 2.0 or greater is considered large (about two *SD*s), and hence we would review the study for any apparent plausible explanation (e.g., the outlier is a parent‐reported measure when all others are teacher‐reported; Zalta, 2011). When a plausible explanation is identified, the outlier will be removed from the analysis. In the event that no explanation is obvious and we can identify no errors in our data entry, we will reduce the extremeness of the outlier (and, therefore, its effect on the average effect size) by setting the effect size to three *SD*s above the mean effect size, including outliers (referred to as either “Winsorizing” or “top‐coding”; Cooper, 2017; Lipsey & Wilson, 2001).

#### 3.6.5 Outcomes

As mentioned earlier, our review is a comprehensive look at the effectiveness of ECE programs across a broad array of outcome measures and ages. To reduce the risk of comparing apples and oranges, we will group outcome measures by age at measurement (using brackets) and then by outcome domain.

We expect to use 10 age brackets: (a) infant/toddler, 0–2 years old; (b) preschool, 3–4 years old; (c) kindergarten, 5–6 years old; (d) first grade, 6–7 years old; (e) second grade, 7–8 years old; (f) third through fifth grade, 8–11 years old; (g) middle school, 11–14 years old; (h) high school, 14–18 years old; (i) young adult, 18–25 years old; and (j) adult, 26 years old and over (we will reconsider these age brackets as we identify studies during the review).

We have identified the following domains and subdomains in which we intend to group effect sizes: (the first eight are identified by the WWC as the primary outcome domains for ECE research (U.S. Department of Education, 2014a), and we would use their definitions when categorizing outcomes).

- Cognitive (general)
- Mathematics
- Social‐emotional development
- Language development
- Alphabetics
- Fluency
- Comprehension
- General reading achievement
- Academic
  - Placement in special education
  - Grade retention
  - Grades/GPA

- Long‐term outcome measures (or, impacts)
  - Educational attainment
  - Crime
  - Income
  - Health, including life expectancy and health behaviors

When a domain comprises subdomains, we will consider combining the effect sizes across the subdomains to generate an overall domain average effect size. The decision to do so will depend on a number of factors, including the number of effect sizes, the type of effect sizes and whether they can be combined (e.g., as mentioned earlier, odds ratios should not be combined with Hedges' *g* effect sizes), and the degree to which the outcomes appear to be sufficiently similar.

#### 3.6.6 Risk of bias

As mentioned above, we will assess the degree of causal validity in each of the included studies and will combine the effect sizes for those rated as *Strong Causal Validity* separately from those rates as *Sufficient Causal Validity*. We will only combine the effect sizes across these groups if the results from our moderator analyses and sensitivity analyses reveal no statistically significant differences in the effect sizes related to this rating.

#### 3.6.7 Combining effect sizes

When a study reports more than one outcome in a given domain (or subdomain) at a particular age, we will average the effect sizes using the inverse‐variance weight function (Dong, Maynard, & Perez‐Johnson, 2008) in order to reduce the likelihood of our results being influenced by “data mining.” We prefer this approach over the WWC's method of using a simple, unweighted average because the inverse‐variance weight accounts for variation in the standard error of each effect size that may be due to differences in sample sizes or measurement error, and therefore seems more accurate.

Likewise, when more than one study reports the same outcomes for the same sample (i.e., they use the same dataset), we will calculate the average effect size across these studies for the common outcomes and use this average in our analysis.

We will then calculate, as is typical practice, the average across‐study effect size for each domain (and subdomain) using random‐effects inverse‐variance weights and will report 95% confidence intervals for all mean effect sizes.

We will consider using robust variance estimation (RVE) if we ultimately perform a meta‐regression and have a sufficient number of studies to apply the method. Hedges, Tipton, and Johnson (2010) caution that RVE “may yield accurate results with as few as 20‐40 studies.” We are therefore reluctant to use this method unless we have upwards of 40 studies.

#### 3.6.8 Assessment of heterogeneity

We expect to find substantial heterogeneity in the programs and populations included in our review, and thus we will use random‐effects meta‐analysis to combine effect sizes and perform moderator analyses in R. Nonetheless, as is common practice in meta‐analysis, we will use the *Q* test to determine whether the level of heterogeneity is statistically significant and the *I* ^2^ statistic to describe the percentage of variation across studies that is due to heterogeneity rather than chance (Higgins & Thompson, 2002; Higgins, Thompson, Deeks, & Altman, 2003).

Random‐effects meta‐analysis requires a measure of between‐study variation, *τ* ^2^, to calculate average across‐study effect sizes, and, although there are many methods for computing *τ* ^2^, each with their own advantages and disadvantages, we expect to use the approach recommended by the Cochrane Statistical Group. This involves using the Paule‐Mandel method to estimate *τ* ^2^ and the Hartung‐Knapp adjustment to calculate confidence intervals for the resulting average across‐study effect sizes (Knapp & Hartung, 2003; Veroniki et al., 2015; Veroniki & Salanti, 2013).

#### 3.6.9 Moderator analyses

Provided we have enough studies and that the information within the studies permits, we will examine the following moderators for their influence on effect sizes either through subgroup analyses using random‐effects assumptions or by using meta‐regression, as detailed by Higgins and Thompson (2002):

- Characteristics of the program (e.g., whether the intervention is a demonstration project, teacher qualifications, use of curriculum, ancillary services provided);
- Duration/intensity of the program (e.g., full‐day vs. half‐day, 12 months vs. academic year);
- Characteristics of the population served (e.g., percent at‐risk, percent English Language Learners, age when receiving services);
- Study design used (e.g., experiment vs. observational study, method of assignment to condition, level of services received by control/comparison group); and
- Characteristics of the study (e.g., study rating [from risk of bias], year the study was conducted, whether the study was published in a peer‐reviewed journal).

We expect that the number of studies that provide effect sizes for a given domain and age bracket will be small, and therefore our ability to conduct these moderator analyses may be limited.

#### 3.6.10 Sensitivity analyses

We will conduct sensitivity analyses to compare the results of studies rated as *Strong Causal Validity* to those rates as *Sufficient Causal Validity* in order to decide whether they should be combined. In addition, we will explore whether and to what extent our decision to Winsorize outlier effect sizes may have affected our findings.

##### Interpreting effect sizes

We plan to use the WWC's improvement index as described in the WWC's *Procedures and Standards* Handbook. We will, however, explore other methods for interpreting our results, including the empirical benchmarks proposed by Hill et al. (2008).

### 3.7 Treatment of qualitative research

Our preliminary searches did not yield any qualitative studies that met our criteria for inclusion, and thus we do not anticipate including many qualitative studies in our review. Nevertheless, should we find more than a couple of eligible qualitative studies, we would employ the method of thematic synthesis to synthesize the findings, which we would use to supplement the results of our meta‐analysis.

Thematic synthesis, as described by Thomas and Harden (2008) and Thomas et al. (2012), is a three‐step process used to identify patterns across qualitative research studies to generate new explanations or hypotheses.

Before beginning the thematic synthesis process, we would gather electronic versions of each study, and if any are unavailable electronically, we would scan these documents and convert them into editable electronic files. Then, as the first step, we would review each study, identify the findings in each, and enter each finding into a database. Each finding would then be individually coded (this is referred to as “line‐by‐line coding”). Next, we would organize these codes, first by grouping and then combining them into “descriptive themes.” Last, we would compare the descriptive themes from each of the studies in order to identify patterns and draw conclusions (referred to as “analytical themes”).

The analytical themes identified using thematic synthesis can be used to supplement gaps in our review that cannot be filled with quantitative analysis. Kluve et al. (2014) and Petrosino et al. (2014) identified overlapping areas where qualitative research can support the quantitative analysis in a systematic review. First, qualitative research can capture program context and nonquantifiable information. Second, qualitative research can illuminate the theory of change or causation of various programs included. Our review would seek to use, if found, qualitative information to fill these gaps that may appear based on our quantitative analysis.

## ROLES AND RESPONSIBLIITIES

- Content: D. B., D. C., and J. S.
- Systematic review methods: D. B., D. C., and J. S.
- Statistical analysis: D. B., D. C., and J. S.
- Information retrieval: D. B., D. C., and J. S.

## SOURCES OF SUPPORT

We received $49,000 from the Jacobs Foundation, $38,080 from an anonymous donor, and support from the University System of Maryland Foundation.

## DECLARATIONS OF INTEREST

We have not been involved with the evaluating of early childhood education programs or in developing a previous systematic review, but we have written a series of assessments of the evaluations of individual early childhood education programs that we have published on our website (http://www.welfareacademy.org/pubs/early_education/index.shtml).

## PLANS FOR UPDATING THE REVIEW

Douglas Besharov is responsible for updating the review. Searches for new primary research will be conducted every other year and updates to the review will be made to reflect new studies accordingly.

## Supporting information

Supporting information

## Footnotes

^1^
The Creative Curriculum is distributed by Teaching Strategies, Inc. and “designed to foster development of the whole child through teacher‐led, small and large group activities centered around 11 interest areas (blocks, dramatic play, toys and games, art, library, discovery, sand and water, music and movement, cooking, computers, and outdoors)” (U.S. Department of Education, 2013).

^2^
This is somewhat akin to the typical program operator selection bias in which the program operator affects an administrative decision (i.e., whether to retain in grade or place in special education) that is also an outcome measure. In the case of child welfare and the decision whether or not to place children in foster care, Heneghan et al. (1996) state: “because workers are unblinded and are readily aware of the treatment being offered to children and their families, placement may be discouraged for children receiving [family preservation services]” (p. 541). Administrative decisions might also be influenced by whether or not an individual has received a particular service rather than whether the program has had a marked effect on that individual (referred to as the “halo effect”).

^3^
The Brookings volume only examines state/district pre‐K programs. It does not consider the evidence from other ECE programs, such as Head Start or demonstration projects like the Perry Preschool Project.

^4^
It should be noted that the Campbell Collaboration library describes three systematic reviews currently underway that may be relevant to our review (Baron, Evangelou, Malmberg, & Melendez‐Torres, 2015; Hjetland, Brinchmann, Lyster, Hagtvet, & Melby‐Lervåg, 2015; Keenan, Connolly, & Stevenson, 2015). Based on the available protocols, however, it appears that the overlap is limited. They either assess much narrower (more specialized) interventions and/or limited aspects of child development.

^5^
Chambers et al. (2010) rate the strength of evidence as: (a) “strong evidence of effectiveness”, (b) “moderate evidence of effectiveness”, (c) “limited evidence of effectiveness: strong evidence of modest effects”, (d) “limited evidence of effectiveness: weak evidence with notable effects”, and (e) “insufficient evidence of effectiveness.” They exclude studies that do not compare one curriculum or programmatic variation to another, and thus do not include any studies for some prominent ECE projects, such as the Abecedarian Project, Building Blocks, and the Perry Preschool Project.

^6^
For example, the children in the Perry Preschool study were selected because their parents were of low socioeconomic status, defined by years of schooling, occupational levels, and rooms per household (Schweinhart et al., 2005). Similarly, researchers of the Abecedarian Project used a “high‐risk index” to select children to participate in the study. The index comprised 13 sociodemographic factors, but only one explicitly measured income and another three were, essentially, indirect measures of income (i.e., payments received from welfare agencies within past 3 years, record of father's work indicates unstable and unskilled or semiskilled labor, and relevant social agencies in the community indicate that the family is in need of assistance; Ramey & Campbell, 1984).

^7^
Immediate (or “short‐term”) outcomes are changes in participants' knowledge, skills, and behaviors that are directly attributable to the program, measured during the program or as close to program completion as possible. Intermediate (or “medium‐term”) outcomes are subsequent changes that result from the immediate outcomes. As such, they should be logically linked to an immediate outcome and might be measured several years after program completion. Impacts are longer‐term consequences of the changes the program made to those subject to it (outcomes) on a specific future aspect of the future prospects (welfare) of individuals, groups, institutions, or communities subject to the intervention (compared with those not) and should be preceded by measured changes in intermediate and immediate outcomes. As such, impacts should be logically linked to one or more intermediate and/or immediate outcomes and, like intermediate outcomes, might be measured many years after participants complete the program (Canadian International Development Agency, 2008; Centers for Disease Control and Prevention, 2007; Centre for Community Based Research, 2005).

^8^
A control variable would be satisfactory if, for example, an evaluation of a math intervention contrasted students' math scores with a measure of their drawing skills. Presumably, math instruction should have no effect on drawing skills, and hence any change in math skills might be attributable to one or more extraneous causes if it is accompanied by a similar change in drawing skills.

^9^
We have decided to limit studies to those of programs in the listed countries for several reasons. First, and most importantly, early childhood programs fit within specific economic, social, familial, and education contexts. It is already a stretch to combine U.S. studies with those from Europe, but we think it can be done appropriately. We do not think, however, that the same can be said outside of the list of countries we provide. A major reason is that the post‐ECE experiences of children are so different that comparisons cannot usefully be made. Second, we do not have the ability to review studies written in any language other than English. Third, restricting studies to those written in English in low‐ or middle‐income countries likely introduces its own bias, as English‐language studies may be systematically different in some form than non‐English studies. Last, we have finite resources to devote to this project. Including studies from every country is cost‐prohibitive, so we made the decision to implement a location restriction that focused on maintaining a minimum threshold for similarity (that is, that the countries share similar cultural and educational contexts).

^10^
We may consider issues related to generalizability and, possibly, general equilibrium during coding and through the use of moderators in our analysis.

^11^
We also consider contamination and implementation weaknesses to be serious threats to causal validity, but we think the best approach is to treat them as moderators rather than as standards or criteria in the study rating process, which is consistent with common practice in other systematic reviews and meta‐analyses. Another concern is the threat posed by possible behavioral responses (i.e., the extent to which members of the program or nonprogram group behave, or perform differently because they know they are in an experimental program), but these tend to be difficult to empirically document, and, indeed, they infrequently are. We think that this threat is most relevant to whether the study evaluates a demonstration project, whether the program was independently evaluated, and/or whether the teachers were blind to the assignment of the children, and thus we will code this information for each study and use as moderators in our analysis.

^12^
Of the threats we are treating as moderators, we think substitution bias might be the most prevalent, but, like the others, it does not appear to be an issue typically examined during the study rating process of systematic reviews, particularly those of ECE programs (some reviews do code this information, e.g., Duncan & Magnuson, 2013; Elango, Garcia, Heckman, & Hojman, 2015). Indeed, after reviewing more than 20 published study rating tools, we found none that consider substitution bias. We think, however, that substitution has the potential to be a serious threat in ECE evaluations, especially given the increased prevalence of child care enrollment among low‐income children in the past 10 years. For example, Besharov and Morrow (2010) estimate the rate of uptake in center‐based child care among poor 4‐year‐olds to be at least 65% in 2003, and the Head Start Impact Study similarly reports that about 60% of children in the control group participated in child care or early education programs, including control group children who received Head Start (Puma, Cook, & Heid, 2010). Using substitution as a criterion, however, would likely have a systematic exclusionary effect on ECE evaluations conducted in the past 10 years. We think, therefore, that the best approach is to treat it as a moderator variable and rate the level of substitution (albeit, somewhat arbitrarily) during the coding process.
