# Supplementary material for: PROTOCOL: Early childhood education programs for improving the development and achievement of low‐income children: a systematic review
Source: Campbell Syst Rev. 2020 Sep 2;16(3):e1100. doi: 10.1002/cl2.1100 (PMC8356309; doi:10.1002/cl2.1100)
Supplement: Supplementary file 1 — Supporting information [file CL2-16-e1100-s001.docx]

**APPENDIXES**

**APPENDIX A**

**Reviews Discussed in the “Prior Reviews” Section**

* *indicates the thirteen systematic reviews for which findings are summarized*

Anderson, L. M., Shinn, C., Fullilove, M. T., Scrimshaw, S. C., Fielding, J. E., Normand, J., . . . & Task Force on Community Preventive Services. (2003). The effectiveness of early childhood development programs: A systematic review. *American Journal of Preventive Medicine, 24*(3), 32–46. doi: 10.1016/S0749-3797(02)00655-4

*Barnett, W. S. (1995). Long-term effects of early childhood programs on cognitive and school outcomes. *The Future of Children, 5*(3), 25–50. doi:10.2307/1602366

Barnett, W. S. (1998). Long-Term Cognitive and Academic Effects of Early Childhood Education on Children in Poverty. *Preventive Medicine, 27*(2), 204–207. doi:10.1006/pmed.1998.0275

Boocock, S. S. (1995). Early Childhood programs in other nations: Goals and outcomes. *The Future of Children, 5*(3), 94–114. doi: 10.2307/1602369

*Burger, K. (2010). How does early childhood care and education affect cognitive development? An international review of the effects of early interventions for children from different social backgrounds. *Early Childhood Research Quarterly, 25*(2), 140–165. <https://doi.org/10.1016/j.ecresq.2009.11.001>

*Camilli, G., Vargas, S., Ryan, S., & Barnett, W. S. (2010). Meta-analysis of the effects of early education interventions on cognitive and social development. *The Teachers College Record, 112*(3), 579–620. <http://www.gregorycamilli.info/papers/early%20education%20interventions.pdf>

Casto, G., & Mastropieri, M. A. (1986). The efficacy of early intervention programs: A meta-analysis. *Exceptional Children, 52*(5), 417–424. <https://doi.org/10.1177/001440298605200503>

Chambers, B., Cheung, A., Slavin, R. E., Smith, D., & Laurenzano, M. (2010)*. Effective early childhood education programmes: A systematic review*. Baltimore, MD: Johns Hopkins University, Center for Research and Reform in Education. <http://www.successforall.org/SuccessForAll/media/PDFs/early_child_ed_Sep_22_2010.pdf>

Chambers, B., Cheung, A. C. K., & Slavin, R. E. (2016). Literacy and language outcomes of comprehensive and developmental-constructivist approaches to early childhood education: A systematic review. *Educational Research Review, 18*, 88–111. <https://doi.org/10.1016/j.edurev.2016.03.003>

Chambers, B., de Botton, O., Cheung, A., & Slavin, R. E. (2012). Effective early childhood education programs for disadvantaged children. In O. N. Saracho & B. Spodek (Eds.), *Handbook of research on the education of young children* (3rd ed., pp. 322–331). New York, NY: Routledge.

Coglan, M., Bergeron, C., White, K., Sharp, C., Morris, M., & Wilson R. (2010). *Narrowing the gap in outcomes for young children through effective practices in the early years*. London: Centre for Excellence and Outcomes in Children and Young People's Services.

Currie, J. (2001). Early childhood education programs. *Journal of Economic Perspectives, 15*(2), 213–238. doi: 10.1257/jep.15.2.213

Darrow, C. L. (2009). Language and literacy effects of curriculum interventions for preschools serving economically disadvantaged children: A meta analysis. *Society for Research on Educational Effectiveness*. <https://eric.ed.gov/?id=ED514949>

*D’Onise, K. McDermott, R. A., Lynch, J. W. (2010). Does Attendance at preschool affect adult health? A systematic review. *Public Health, 124*(9), 500–511. doi:10.1016/j.puhe.2010.05.004

*D’Onise, K., Lynch, J. W., Sawyer, M. G., & McDermott, R. A. (2010). Can preschool improve child health outcomes? A systematic review. *Social Science and Medicine, 70*, 1423–1440. <https://doi.org/10.1016/j.socscimed.2009.12.037>

Duncan, G. J., Ludwig, J. & Magnuson, K. A. (2007). Reducing poverty through preschool interventions. *The Future of Children, 17*(2), 143–160. Retrieved from <http://files.eric.ed.gov/fulltext/EJ795866.pdf>

*Duncan, G. J., & Magnuson, K. A. (2013). Investing in preschool programs. *Journal of Economic Perspectives, 27*(2), 109–132. doi: 10.1257

Elango, S., García, J. L., Heckman, J. J. & Hojman, A. (2015). Early childhood education (No. 21766). *National Bureau of Economic Research*. <http://www.nber.org/papers/w21766.pdf>

Fryer, R. G. (2016). The production of human capital in developed countries: Evidence from 196 randomized field experiments. In A. V. Banerjee & E. Duflo (Eds.), *Handbook of economic field experiments* (vol. 2, pp. 95–322). North-Holland. <https://doi.org/10.1016/bs.hefe.2016.08.006>

Fukkink, R., Jilink, L., & Oostdam, R. (2017). A meta-analysis of the impact of early childhood interventions on the development of children in the Netherlands: An inconvenient truth? *European Early Childhood Education Research Journal,25*(5), 656–666. <https://doi.org/10.1080/1350293X.2017.1356579>

*Gilliam, W. S. & Zigler, E. F. (2000). A critical meta-analysis of all evaluation of state-funded preschool from 1977 to 1998: Implication for policy, service delivery and program evaluation. *Early Childhood Research Quarterly, 14*(4), 441–473. <https://doi.org/10.1016/S0885-2006(01)00073-4>

*Gorey, K. M. (2001). Early childhood education: A meta-analytic affirmation of the short- and long-term benefits of educational opportunity. *School Psychology Quarterly, 16*(1), 9–30. <http://scholar.uwindsor.ca/cgi/viewcontent.cgi?article=10>

Grindal, T., Bowne, J. B., Yoshikawa, H., Schindler, H. S., Duncan, G. J., Magnuson, K., & Shonkoff, J. P. (2016). The added impact of parenting education in early childhood education programs: A meta-analysis. *Children and Youth Services Review, 70*, 238–249. <https://doi.org/10.1016/j.childyouth.2016.09.018>

Hall, A. H., Simpson, A., Guo, Y., & Wang, S. (2015). Examining the effects of preschool writing instruction on emergent literacy skills: A systematic review of the literature. *Literacy Research and Instruction, 54*(2), 115–134. <https://doi.org/10.1080/19388071.2014.991883>

Karoly, L. A., Kilburn, M. R., & Cannon, J. S. (2005). *Early childhood interventions: Proven results, future promise*. Santa Monica, CA: RAND Corporation.

Karoly, L. A. (2016). The economic returns to early childhood education. *Future of Children, 26*(2), 37–55. doi: 10.1353/foc.2016.0011

*Kay, N., & Pennucci, A. (2014). *Early childhood education for low-income students: A review of the evidence and benefit-cost analysis*. Olympia, WA: Washington State Institute for Public Policy. Retrieved from [https://www.wsipp.wa.gov/ReportFile/1547/Wsipp_Early-Childhood-Education-for-Low-Income-Students-A-Review-of-the-Evidence-and-Benefit-Cost-Analysis_Full-Report.pdf](https://www.wsipp.wa.gov/ReportFile/1547/Wsipp_Early-Childhood-Education-for-Low-Income-Students-A-Review-of-the-Evidence-and-B)

Magnuson, K. A., & Waldfogel, J. (2005). Early childhood care and education: Effects on ethnic and racial gaps in school readiness. *The Future of Children, 15*(1), 169–196. Retrieved from <https://www.jstor.org/stable/1602667>

*Manning, M., Homel, R., & Smith, C. (2010). A meta-analysis of the effects of early developmental prevention programs in at-risk populations on non-health outcomes in adolescence. *Children and Youth Services Review, 32*, 506–519. <https://doi.org/10.1016/j.childyouth.2009.11.003>

*McCoy, D. C., Yoshikawa, H., Ziol-Guest, K. M., Duncan, G. J., Schindler, H. S., Magnuson, K., Yang, R., Koepp, A., & Shonkoff, J. P. (2017). Impacts of early childhood education on medium- and long-term educational outcomes. *Educational Researcher, 46*(8), 474–487. <https://doi.org/10.3102/0013189X17737739>

*McKey, R.H. (1985). *The impact of Head Start on children, families and communities: Final report of the Head Start Evaluation, Synthesis and Utilization Project*. Washington, DC: Administration for Children, Youth, and Families, U.S. Department of Health and Human Services. <http://files.eric.ed.gov/fulltext/ED263984.pdf>

Melhuish, E., Ereky-Stevens, K., Petrogiannia, K., Ariescu, A., Penderi, E., Rentzou, K., Tawell, A., Leseman, P., & Broekhuisen, M. (2015). *A review of research on the effects of early childhood education and care (ECEC) on child development*. Brussels: European Commission. <http://ecec-care.org/fileadmin/careproject/Publications/reports/CARE_WP4_D4__1_review_of_effects_of_ecec.pdf>

Mitchell, L., Wylie, C., Carr, M. (2008). *Outcomes of early childhood education: Literature review*. Wellington N.Z.: New Zealand Council for Educational Research. <http://www.nzcer.org.nz/system/files/885_Outcomes.pdf>

*Nelson, G., Westhues, A., & MacLeod, J. (2003). A meta-analysis of longitudinal research on preschool prevention programs for children. *Prevention & Treatment, 6*, 1–35. <http://dx.doi.org/10.1037/1522-3736.6.1.631a>

Phillips, D. A., Lipsey, M. A., Dodge, K. A., Haskins, R., Bassok, D., Burchinal, M. A., Duncan, G. J., Dynarksi, M., Magnuson, K. A., & Weiland, C. (2017). *The current state of scientific knowledge on pre-kindergarten effects.* Washington, DC: Brookings Institution. <https://www.brookings.edu/wp-content/uploads/2017/04/duke_prekstudy_final_4-4-17_hires.pdf>

Reynolds, A. J., Magnuson, K. A., & Ou, S. R. (2010). Preschool-to-third grade programs and practices: A review of research. *Children and Youth Services Review, 32*, 1121–1131. <https://doi.org/10.1016/j.childyouth.2009.10.017>

Ulferts, H., & Anders, Y. (2015). *Effects of ECEC on academic outcomes in literacy and mathematics: A meta-analysis of European longitudinal studies*. Brussels: European Commission. <http://ecec-care.org/fileadmin/careproject/Publications/reports/CARE_WP4_D4_2_Metaanalysis_public.pdf> (accessed 1/8/2019)

van Huizen, T. & Plantenga, J. (2018). Universal child care and children's outcomes: A meta-analysis of evidence from natural experiments. *Economics of Education Review, 66*, 206–222. <https://doi.org/10.1016/j.econedurev.2018.08.001>

Vandell, D. L. & Wolfe, B. (2000). *Child care quality: Does it matter and does it need to be improved?* Madison, WI: Institute for Research on Poverty. <https://pdfs.semanticscholar.org/4722/d210ea739a7c0eae6de63f55eccb57a27671.pdf>

White, K. R. (1985). Efficacy of early intervention. *Journal of Special Education, 19*(4), 401–416. <https://doi.org/10.1177/002246698501900405>

White, K. R., Taylor, M. J., & Moss, V. D. (1992). Does research support claims about the benefits of involving parents in early intervention programs? *Review of Educational Research, 62*(1), 91–125. <https://doi.org/10.3102/00346543062001091>

Yoshikawa, H. (1995). Long-term effects of early childhood programs on social outcomes and delinquency. *The Future of Children, 5*(3), 51–75. doi: 10.2307/1602367

Yoshikawa, H., Weiland, C., Brooks-Gunn, J., Burchinal, M. R., Espinosa, L. M., Gormley, W. T., . . . & Zaslow, M. J. (2013). *Investing in our future: The evidence base on preschool education*. New York, NY and Ann Arbor, MI: Foundation for Child Development and Society for Research in Child Development.

**APPENDIX B**

**Findings from Thirteen Systematic Reviews**

Despite the limitations described above, we think it important to summarize these studies in order to understand and assess our own findings and how they may either support or contradict earlier research. We, therefore, briefly summarize what this body of thirteen “systematic” research reviews says about the effects of ECE participation on children’s cognitive (e.g., IQ and achievement test scores and grades),^[[1]](#footnote-1)^ socio-emotional, behavioral, and health outcomes, both in the short- and long-term. In addition, we discuss what they say about possible differential effects due to participant, program, and/or study characteristics.

*Cognitive*

Overall, these reviews find that, on average, ECE programs have a positive effect on children’s test scores, with marked improvement immediately after the intervention. (See table B.1 for a listing of cognitive outcomes by study.) This immediate effect, however, appears to “fade out” as children age and progress through school. For example, in their analysis of cognitive effects, Kay and Pennucci (2014) synthesized standardized test score results from fifty-nine studies, finding an average immediate effect size of *d* = 0.31 that diminishes to *d* = 0.15 between kindergarten and second grade, *d* = 0.10 between third and fifth grade, and *d* = 0.09 between sixth and ninth grade. This “fade out” is also reported by Camilli, Vargas, Ryan, and Barnett (2010); Gilliam and Zigler (2000); McKey et al. (1985); and Nelson, Westhues, and MacLeod (2003).

Although cognitive effects appear to fade out, there may be some positive effects on grade retention, placement in special education, and high school graduation. In their review of 123 studies, Camilli et al. (2010) utilized two “contrast groups”: a treatment versus no treatment contrast and a treatment versus alternative contrast. For the former, they found a statistically significant effect size of *ES* = 0.14 for the “school progress domain,” which includes “school grades, academic track, special education placement, high school completion, and college attendance,” (Camilli et al., p. 592) but, notably, not grade retention. This effect on the school progress domain, however, was only statistically significant when treatment children were compared to a no service group (the first contrast group).

Similarly, Manning, Homel, and Smith (2010) reviewed seventeen studies investigating medium- and long-term effects of ECE programs, finding that ECE participation had a statistically significant, positive effect (*d* = 0.53) on “education success,” an outcome which includes: “Special education; feeling of belonging at school; graduation; school drop-out; long-term school suspension; grade retention; completed years of education; school attendance (e.g. > 20 absent days from school per year); [and] learning problems” (p. 510).

McCoy et al. (2017), Kay and Penucci (2014), and Gorey (2001) also find reductions in the rate of grade retention and placement in special education.

Some reviews, however, report less optimistic results on these outcomes. In his review of U.S. and international programs, Burger (2010) finds that only two of six studies report reductions in either grade retention or special education placement and only one of two studies found a slight increase in the number of years of school completed.^[[2]](#footnote-2)^ Likewise, in their review of thirteen state ECE programs, Gilliam and Zigler (2000) find that most of the effect sizes for grade retention and special education (including both referral to and placement in special education outcomes) were not statistically significant. (They do not report on high school graduation.)

***Table B.1: Cognitive Outcomes Reported in Thirteen Systematic Reviews (By Study)***

| **Study and types of programs included**  **(from most to least recent)** | **Outcome** | **Key finding(s) (standard error (SE)/standard deviation/p-value)** | **# of studies, effect sizes (ES)/estimates^1^ synthesized and/or considered** | **Limitation(s)^2^** |
| --- | --- | --- | --- | --- |
| McCoy et al. (2017)  *Head Start, state/district, and model* | Grade retention | *d* = -0.33 (p < 0.01) | 39 ES | Not up-to-date, focused only on long-term outcomes |
|  | Special education | *d* = -0.26 (p < 0.01) | 19 ES |  |
|  | High school graduation | *d* = 0.24 (p < 0.01) | 17 ES |  |
| Kay and Pennucci (2014)  *Head Start, state/district, and model*  Kay and Pennucci (2014) continued | Test scores  End of pre-K  K-2nd grade  3rd-5th grade  6th-9th grade | *d* = 0.31 (0.03)  *d* = 0.15 (0.02)  *d* = 0.10 (0.01)  *d* = 0.09 (0.03) | 37 ES  38 ES  29 ES  12 ES | Possible apples and oranges problem, not up-to-date |
|  | Age 4 test scores  Head Start  State/district programs  Model programs | *d* = 0.17 (0.03)  *d* = 0.32 (0.03)  *d* = 0.57 (0.12) | 7 ES  17 ES  3 ES |  |
|  | Grade retention  Head Start  State/district programs  Model programs | *d* = -0.08 (0.13)  *d* = -0.39 (0.09)  *d* = -0.46 (0.25) | 5 ES  4 ES  3 ES |  |
|  | Special education placement  Head Start  State/district programs  Model programs | *-*  *d* = -0.23 (0.14)  *d* = -0.47 (0.26) | -  3 ES  3 ES |  |
|  | High school graduation  Head Start  State/district programs  Model programs | *d* = 0.08 (0.08)  *d* = 0.18 (0.14)  *d* = 0.31 (0.27) | 2 ES  2 ES  3 ES |  |
| Duncan and Magnuson (2013)  *Head Start, state/district, and model* | Cognitive and achievement score | *d* = 0.35 (no SE or statistical significance data reported) | 84 ES | Not up-to-date, focused only on immediate cognitive outcomes |
| Burger (2010)  *U.S. and non-U.S., Head Start, state/district, and model* | Cognitive outcomes^3^ | ^“^Mostly positive” in 22 studies (statistical significance is unclear) | 32 studies | Not up-to-date (and excludes studies conducted before 1995) |
|  | Grade retention | 2 beneficial effect estimates (we presume, p < 0.05)^4^ | 7 effect estimates |  |
|  | Special education | 2 beneficial effect estimates (p < 0.05) | 8 effect estimates |  |
|  | High school graduation, college attendance, and educational attainment | 5 beneficial effects estimates (we presume, p < 0.05)^4^ | 10 effect estimates |  |
| Camilli, Vargas, Ryan, and Barnett (2010)^5^  *Head Start, state/district, and model* | Cognitive, overall | simple average ES = 0.23 (p < 0.01) | 306 ES | Possible apples and oranges problem, not up-to-date |
|  | School progress | simple average ES = 0.14 (p < 0.01) | 60 ES |  |
| Manning, Homel, and Smith (2010)  *State/district and model* | Cognitive development (at adolescence) | *d* = 0.34 (p < 0.01) | 14 studies | Possible apples and oranges problem, not up-to-date, focused only on intermediate outcomes |
|  | Educational success (at adolescence) | *d =*  0.53 (p < 0.01) | 10 studies |  |
| Nelson, Westhues, and MacLeod (2003)  *U.S. and non-U.S.; Head Start, state/district, and model* | Cognitive, overall  Measured in preschool  Measured in K-8 | *d =* 0.52 (no SE or statistical significance data reported)  *d =* 0.30 (no SE or statistical significance data reported) | 17 studies  27 studies | Possible apples and oranges problem, not up-to-date |
| Gorey (2001)  *Head Start and model* | Intelligence | *U_3_* = 76.5 (p > 0.05) | 23 ES | Possible apples and oranges problem, not up-to-date |
|  | Academic achievement | *U_3_* = 78.2 (p > 0.05) | 17 ES |  |
|  | Grade retention | RR = 0.52 (p < 0 .05) | 8 ES |  |
|  | Not a high school graduate | RR = 0.54 (p < 0.05) | 7 ES |  |
| Gilliam and Zigler (2000)  *State/district only*  Gilliam and Zigler (2000) continued | Achievement tests  Kindergarten  1st grade  2nd grade  3rd grade  4th grade | (at p < 0.05)  2 positive ES  3 positive ES  1 negative ES  0 statistically significant  5 positive ES  2 negative ES | 2 ES  10 ES  6 ES  14 ES  2 ES | Not up-to-date |
|  | Grades  Kindergarten  1st grade  2nd grade  3rd grade  4th grade | (at p < 0.05)  2 positive ES  1 positive ES  0 statistically significant  2 negative ES  0 statistically significant | 6 ES  8 ES  4 ES  6 ES  4 ES |  |
|  | Grade retention  Kindergarten  1st grade  2nd grade  3rd grade  4th grade | (at p < 0.05)  2 beneficial ES  2 beneficial ES  1 beneficial ES  1 adverse ES  2 beneficial ES  1 adverse ES  0 statistically significant | 3 ES  15 ES  4 ES  5 ES  2 ES |  |
|  | Special education (both placement and referral)  Kindergarten  1st grade  2nd grade  3rd grade  4th grade | (at p < 0.05)  0 statistically significant  1 beneficial ES  2 beneficial ES  0 statistically significant  0 statistically significant | 6 ES  6 ES  5 ES  3 ES  2 ES |  |
|  | Perceived competence  End of pre-K  Kindergarten  1st grade | (at p < 0.05)  1 positive ES  0 statistically significant  0 statistically significant | 1 ES  1 ES  1 ES |  |
| Barnett (1995)  *Head Start, state/district, and model*  Barnett (1995) continued | Achievement  Model programs  Large-scale programs | (at p < 0.05)  3 studies with positive effects  5 studies with positive effects  7 studies with mixed effects | 11 studies  21 studies | Not up-to-date, focused only on long-term outcomes |
|  | Grade retention and special education  Model programs  Large-scale programs | (at p < 0.05)  2 studies with beneficial effects  5 studies with beneficial effects  2 studies with adverse effects | 13 studies  11 studies |  |
|  | Special education  Model programs  Large-scale programs | (at p < 0.05)  3 studies with beneficial effects  1 study with adverse effects  3 studies with beneficial effects | 12 studies  8 studies |  |
|  | High school graduation  Model programs  Large-scale programs | (at p < 0.05)  1 study with positive effects  1 study with positive effects | 3 studies  1 study |  |
| McKey et al. (1985)  *Head Start only* | Intelligence tests  Immediate  1 year after  2 years after  ≥3 years after | *d* = 0.59 (0.42)  *d* = 0.09 (0.40)  *d* = -0.03 (0.28)  *d* = -0.20 (0.25) | 19 ES  17 ES  17 ES  11 ES | Not up-to-date |
|  | School readiness tests  Immediate  1 year after  2 years after | *d* = 0.31 (0.37)  *d* = 0.21 (0.27)  *d* = 0.02 (0.31) | 13 ES  10 ES  11 ES |  |
|  | Achievement tests  Immediate  1 year after  2 years after  ≥3 years after | *d =* 0.54 (0.51)  *d =* 0.20 (0.22)  *d =* 0.13 (0.41)  *d =* 0.00 (0.21) | 7 ES  7 ES  15 ES  22 ES |  |
| *Note:* ^1^ We use the term “effect estimate” when insufficient information is provided in the review to determine whether or not the estimate is a formal effect size; *^2^* Limitations are discussed above (see section on “limitations of these reviews”); ^3^ Based on the data in Burger’s (2010) Table 2, it is unclear how many effect estimates are reported, so we report the number of studies instead; ^4^ The notes in Burger’s (2010) Table 3 do not indicate statistical significance, but, based on the coding used in Table 2, we presume that the same applies here; ^5^ Camilli et al. (2010) report effects for two different “contrast” groups, a treatment versus no treatment contrast and a treatment versus alternative contrast—we report findings only for the former; - indicates no outcome reported; *d*, is standardized mean difference (including Cohen’s *d* and Hedges’ *g*); RR is risk ratio; *U_3_*, is Cohen’s *U_3_* statistic. | | | | |

*Socio-emotional and behavioral*

There is much less consensus on the effects of ECE participation on socio-emotional and behavioral outcomes. Some studies find overall positive effects. (See table B.2 for a listing of socio-emotional and behavioral outcomes by study.) For example, Nelson, Westhues, & MacLeod (2003) report average socio-emotional effect sizes from kindergarten through eight grade (*d* = 0.27) and also from high school and beyond (*d* = 0.33). Manning et al. (2010) also report positive effects on socio-emotional outcomes. They find average effect sizes of *d* = 0.16 and *d* = 0.37 for “social emotional development” and “social participation,” respectively.

Others, however, find mostly inconclusive evidence or no effect. For example, D’Onise et al. (2010a) examined the short-term effects of ECE programs on a few socio-emotional outcomes, namely self-concept, self esteem, social competence, internalizing problems, and externalizing problems. Of the 123 effect estimates (i.e., effect sizes, risk ratios, absolute risk differences) they include, only 25 (20.3 percent) were beneficial, statistically significant (at the 0.05 significance level), and “clinically relevant.” On the other hand, 15 (12.2 percent) were statistically significant, “clinically relevant” adverse effects.^^[[3]](#footnote-3)^1^

Likewise, Gilliam and Zigler (2000) and McKey et al. (1985) find wide variation in the socio-emotional outcomes they examine.

***Table B.2: Socio-emotional and Behavioral Outcomes Reported in Thirteen Systematic Reviews (By Study)***

| **Study, and types of programs included**  **(from most to least recent)** | **Outcome** | **Key finding(s) (standard error (SE)/standard deviation/p-value)** | **# of studies, effect sizes (ES) synthesized and/or considered** | **Limitation(s)^1^** |
| --- | --- | --- | --- | --- |
| Kay and Pennucci (2014)  *Head Start, state/district, and model* | Self-regulation  Head Start  State/district programs  Model programs | *d* = 0.16 (0.13)  *d* = 0.21 (0.04)  - | 1 ES  4 ES | Possible apples and oranges problem, not up-to-date |
|  | Emotional development  Head Start  State/district programs  Model programs | *d* = 0.03 (0.05)  *d* = 0.04 (0.06)  - | 2 ES  5 ES |  |
| Camilli, Vargas, Ryan, and Barnett (2010)^2^  *Head Start, state/district, and model* | Social, overall | Unweighted average ES = 0.16  (p < 0 .01) | 113 ES | Possible apples and oranges problem, not up-to-date |
| D’Onise, Lynch, Sawyer, and McDermott (2010a)  *U.S. and non-U.S.; Head Start, state/district, and model*  D’Onise, Lynch, Sawyer, and McDermott (2010a) continued | Self-esteem | 0 beneficial/adverse ES | 5 ES | Not up-to-date, focused only on short- and intermediate-term outcomes |
|  | Self-concept | 1 beneficial ES (p < 0.05 and “clinically relevant”) | 4 ES |  |
|  | Social competence | 10 beneficial ES (p < 0.05 and “clinically relevant”)  4 adverse ES (p < 0.05 and “clinically relevant”) | 49 ES |  |
|  | Internalizing behaviors | 1 beneficial ES (p < 0.05 and “clinically relevant”) | 5 ES |  |
|  | Externalizing behaviors | 13 beneficial ES (p < 0.05 and “clinically relevant”)  9 adverse ES (p < 0.05 and “clinically relevant”) | 60 ES |  |
| D’Onise, McDermott, and Lynch (2010b)  *U.S. and non-U.S.; Head Start, state/district, and model* | Self-esteem | 0 beneficial/adverse ES (p < 0.05 and “clinically relevant”) | 4 ES | Not up-to-date, focused only on long-term outcomes |
| Manning, Homel, and Smith (2010)  *State/district and model* | Social-emotional development (at adolescence) | *d =* 0.16 (p < 0.05) | 6 studies | Possible apples and oranges problem, not up-to-date, focused only on intermediate outcomes |
|  | Social participation | *d =* 0.37 (p < 0.01) | 3 studies |  |
|  | Deviance (at adolescence) | *d =* 0.48 (p < 0.01) | 3 studies |  |
| Gilliam and Zigler (2000)  *State/district only* | Behavior problems  End of pre-K  Kindergarten  1st grade  2nd grade  3rd grade  4th grade | (at p < 0.05)  1 positive ES  0 statistically significant  1 negative ES  1 negative ES  1 negative ES  1 positive ES | 3 ES  5 ES  5 ES  5 ES  3 ES  2 ES | Not up-to-date |
| Nelson, Westhues, and MacLeod (2003)  *U.S. and non-U.S.; Head Start, state/district, and model* | Socio-emotional, overall  Measured in K-8  Measured in high school and beyond | *d =* 0.27 (no SE or statistical significance data reported)  *d =* 0.33 (no SE or statistical significance data reported) | 19 studies  10 studies | Possible apples and oranges problem, not up-to-date |
| McKey et al. (1985)  *Head Start only* | Self-esteem  Immediate  1 year after  2 years after  ≥3 years after | *d =* 0.17 (0.79)  *d* = -0.20 (0.26)  *d* = 0.01 (0.19)  *d* = -0.14 (0.00) | 3 ES  3 ES  3 ES  1 ES | Not up-to-date |
|  | Achievement motivation  Immediate  1 year after  2 years after  ≥3 years after | *d* = 0.22 (0.28)  *d* = -0.11 (0.52)  *d* = 0.06 (0.28)  *d* = 0.08 (0.18) | 10 ES  15 ES  8 ES  5 ES |  |
|  | Social behavior  Immediate  1 year after  2 years after  ≥3 years after | *d* = 0.35 (0.27)  *d =* 0.16 (0.20)  *d =* 0.63 (1.12)  *d =* -0.10 (0.14) | 5 ES  4 ES  2 ES  2 ES |  |
| *Note:* *^1^* Limitations are discussed above (see section on “limitations of these reviews”); ^2^ Camilli et al. (2010) report effects for two different “contrast” groups, a treatment versus no treatment contrast and a treatment versus alternative contrast, we report findings only for the former; - indicates no outcome reported; *d*, is standardized mean difference (including Cohen’s *d* and Hedges’ *g*). | | | | |

*Health*

Few reviews examine health outcomes either during childhood or adulthood, and, among those that do, they generally find little-to-no evidence of an effect. (See table B.3 for a listing of health outcomes by study.) The strongest evidence comes from two complementary, well-conducted systematic reviews by D’Onise and colleagues (D’Onise et al. 2010a; and D’Onise et al., 2010b). D’Onise et al. (2010a) examine (excluding those soocio-emotional outcomes mentioned above) sixty-five effect estimates across nine child health outcomes, including physical and mental health. Of these, they find that only fifteen (23.1 percent) were beneficial, statistically significant (at the 0.05 significance level), and “clinically relevant.” (The rest showed no effect or no statistically significant, “clinically relevant” effect.) D’Onise et al. (2010b) examined eighty-eight effect estimates (excluding those socio-emotional outcomes we discuss above), of which eight (9.1 percent) were beneficial and statistically significant (at the 0.05 significance level) and one (1.1 percent) was adverse and statistically significant.^[[4]](#footnote-4)^

***Table B.3: Health Outcomes Reported in Thirteen Systematic Reviews (By Study)***

| **Study, and types of programs included**  **(from most to least recent)** | **Outcome** | **Key finding(s) (standard error (SE)/standard deviation/p-value)** | **# of studies, effect sizes (ES) synthesized and/or considered** | **Limitation(s)^1^** |
| --- | --- | --- | --- | --- |
| D’Onise, Lynch, Sawyer, and McDermott (2010a)  *U.S. and non-U.S.; Head Start, state/district, and model* | Obesity, growth, and diet | 8 beneficial ES (p < 0.05 and “clinically relevant”) | 21 ES | Not up-to-date, focused only on short- and intermediate-term outcomes |
|  | Health service use | 2 beneficial ES (p < 0.05 and “clinically relevant”) | 10 ES |  |
|  | Fitness | 0 beneficial/adverse ES (p < 0.05 and “clinically relevant”) | 2 ES |  |
|  | Drug and alcohol | 0 beneficial/adverse ES (p < 0.05 and “clinically relevant”) | 3 ES |  |
|  | Illness, asthma | 0 beneficial/adverse ES (p < 0.05 and “clinically relevant”) | 5 ES |  |
|  | Preventive behaviors, injury | 0 beneficial/adverse ES (p < 0.05 and “clinically relevant”) | 4 ES |  |
|  | Immunization | 4 beneficial ES (p < 0.05 and “clinically relevant”) | 12 ES |  |
|  | General Health | *1 beneficial ES (p < 0.05 and “clinically relevant”)* | *6 ES* |  |
| D’Onise, McDermott, and Lynch (2010b)  *U.S. and non-U.S.; Head Start, state/district, and model* | Blood pressure | 0 beneficial/adverse ES (p < 0.05 and “clinically relevant”) | 4 ES | Not up-to-date, focused only on long-term outcomes |
|  | Overweight and obesity | 3 beneficial ES (p < 0.05 and “clinically relevant”) | 8 ES |  |
|  | Chronic disease diagnosis | 0 beneficial/adverse ES (p < 0.05 and “clinically relevant”) | 3 ES |  |
|  | General health | 0 beneficial/adverse ES (p < 0.05 and “clinically relevant”) | 7 ES |  |
|  | Mental health/illness | 3 beneficial ES (p < 0.05 and “clinically relevant”) | 9 ES |  |
| Gilliam and Zigler (2000)  *State/district only* | Behavior problems  1st grade  2nd grade  3rd grade | (at p < 0.05)  0 statistically significant  0 statistically significant  0 statistically significant | 1 ES  1 ES  1 ES | Not up-to-date |
| *Note:* *^1^* Limitations are discussed above (see section on “limitations of these reviews”). | | | | |

*Crime, teen pregnancy, and economic impacts*

Only three reviews (i.e., Gorey, 2001; Manning et al., 2010; and Key & Pennuchi, 2014) look at the effect of ECE participation on impacts (e.g., crime, teen pregnancy, use of welfare, and employment and earnings),^[[5]](#footnote-5)^ perhaps because only a handful of studies have followed children into adulthood (e.g., Perry Preschool, Abecedarian Project, and Chicago Child-Parent Center) or used large datasets, such as the NLSY, to estimate these impacts (e.g., Garces, Thomas, & Currie, 2003; and Deming, 2009). Overall, these reviews report beneficial effects on crime, teen pregnancy, use of welfare, and employment and earnings. (See table B.4 for a listing of these outcomes/impacts by study.)

All three examined crime outcomes, finding positive overall effects. Gorey (2001) and Manning et al. (2010) calculated effect sizes, finding statistically significant beneficial effects.^[[6]](#footnote-6)^ A more recent review by Key and Pennuchi (2014) however, reports non-statistically significant effect sizes that are based on similar studies and are similar in magnitude to those found by Gorey (2001) and Manning et al. (2010). This might suggest that these results are very sensitive to the meta-analytic procedures and/or the inclusion/exclusion of one or more studies.

Two reviews examined teen pregnancy, specifically, both finding beneficial effects. Gorey (2001) finds that ECE participation reduces teen pregnancy by 25 percent (p < 0.05) and Key and Pennuchi (2014) find effect sizes for Head Start programs of d = -0.47 and for model programs of d = -0.44, although these effects are not significant at the 0.05 significance level.

Gorey (2001) reports economic impacts, finding overall positive effects, although the results, like those for crime, are based on only a handful of studies. Specifically, he finds that children who participated in ECE are 61 percent less likely to be on welfare, 26 percent less likely to have been on welfare as an adult, 27 percent less likely to be unemployed, 33 percent less likely to have earnings below the “poverty criterion,” and 27 percent less likely to not own a home. (All of these results are statistically significant at the 0.05 significance level.)

***Table B.4: Crime, Teen Pregnancy, and Economic Impacts Reported in Thirteen Systematic Reviews (By Study)***

| **Study, and types of programs included**  **(from most to least recent)** | **Outcome** | **Key finding(s) (standard error (SE)/standard deviation/p-value)** | **# of studies, effect sizes (ES) synthesized and/or considered** | **Limitation(s)^1^** |
| --- | --- | --- | --- | --- |
| Kay and Pennucci (2014)  *Head Start, state/district, and model* | Crime  Head Start  State/district programs  Model programs | *d* = -0.18 (0.27)  *d* = -0.25 (0.17)  *d* = -0.32 (0.21) | 2 ES  1 ES  2 ES | Possible apples and oranges problem, not up-to-date |
|  | Teen births (under age 18)  Head Start  State/district programs  Model programs | *d* = -0.47 (0.29)  -  *d* = -0.44 (0.40) | 1 ES  -  2 ES |  |
| Manning, Homel, and Smith (2010)  *State/district and model* | Criminal justice (at adolescence) | *d =* 0.24 (p < 0.001) | 5 studies | Possible apples and oranges problem, not up-to-date, focused only on intermediate outcomes |
| Gorey (2001)  *Head Start and model* | Welfare dependence  Currently receives welfare  Ever received welfare as adult | RR = 0.39 (p < 0 .05)  RR = 0.74 (p < 0 .05) | 2 ES  1 ES | Possible apples and oranges problem, not up-to-date |
|  | Economic well-being  Currently unemployed  Earning below poverty criterion  Not a home owner | RR = 0.73 (p < 0 .05)  RR = 0.67 (p < 0 .05)  RR = 0.73 (p < 0 .05) | 2 ES  2 ES  1 ES |  |
|  | Delinquent and criminal behavior  Ever engaged in delinquent  behavior  Ever arrested  Arrested > 5 times  Teen pregnancy | RR = 0.41 (p < 0 .05)  RR = 0.47 (p < 0 .05)  RR = 0.18 (p < 0 .05)  RR = 0.75 (p < 0 .10) | 3 ES  4 ES  1 ES |  |
|  |  |  | 1 ES |  |
| *Note:* *^1^* Limitations are discussed above (see section on “limitations of these reviews”); - indicates no outcome reported*; d*, is standardized mean difference (including Cohen’s *d* and Hedges’ *g*); RR is risk ratio. | | | | |

***Possible differential effects***

Eight reviews (i.e., Barnett, 1995; Burger, 2010; Camilli et al., 2010; Duncan & Magnuson, 2013; Gorey, 2001; Manning et al., 2010; McKey et al., 1985; and Nelson et al., 2003) use data obtained during the study coding process on participant, program, and/or study characteristics to estimate what factors appear to be related to the magnitude of the effect of ECE on children’s outcomes.

*Participant characteristics*

There is no clear evidence of differential effects based on participant characteristics. Barnett (1995) finds that, “in general, there do not appear to be large variations in effects for children from low-income families” (p. 41). The one exception he points to is gender, finding larger effects on achievement tests and graduation rates for girls for some studies, but he cautions that “none of the large-scale studies which explicitly tested for gender differences found any” (p. 41). (Barnett does not list what other participant characteristics he considered.) Likewise, Burger (2010) finds that low-income children do not appear to benefit more than their more affluent counterparts.

Camilli et al. (2010) examine the child’s age and the “percentage of low-income families in the group,” finding no statistically significant differences. Burger (2010) and McKey et al. (1985) also find inconclusive evidence that beginning ECE at a younger age leads to larger benefits.

Nelson et al. (2003) find significantly larger effect sizes for programs that target black children and families, but it is not clear what (or how many) other characteristics they considered.

*Program characteristics*

It is unclear what program characteristics appear to produce larger effects. For program duration, intensity, and provision of follow-on services, there is little consistency in the findings across these eight reviews. For example, both Gorey (2001) and Nelson et al. (2003) find that longer (greater than one year) and more intense programs produce greater effect sizes (in both cases the differences are statistically significant). However, Camilli et al. (2010) also investigate this relationship, finding no statistically significant association, and McKey et al. (1985) find no relationship between program intensity and magnitude of the effect size.

Nelson et al. (2003) find that cognitive effect sizes for programs with a follow-on component are significantly larger than for those without it. Barnett’s (1995) findings, however, are less conclusive. Using results from two studies, he finds one instance where the effect is larger and one without a discernible difference.

There does appear to be some agreement that programs that provide direct instruction (DI), which involves “teachers explicitly instructing children in academic skills and procedures” (Camilli et al., 2010, p. 598), produce larger effects. Both Camilli et al. (2010) and Nelson et al. (2003) consider DI, finding that cognitive outcomes are greater when programs provide this type of explicit instruction.

One study (Camilli et al., 2010) investigates whether the provision of ancillary services leads to larger effects. Surprisingly, Camilli et al. (2010) find that “provision of additional services showed a strong and negative effect on the cognitive domain, and this effect was design consistent” (p. 598). One possible explanation they propose is that “those who received additional services had lower dose levels and longer durations of treatment. . . . Also, the additional service variable correlates negatively with DI, which has a positive impact on the cognitive outcomes” (p. 598).

*Study characteristics*

Overall, there is little evidence that ECE effects vary by study characteristics, with one exception. Duncan and Magnuson (2013) find that “the effect sizes of programs designed by researchers (.39 standard deviations) were significantly larger than programs not designed by researchers (.18 standard deviations” (p. 114). (They also find that effect sizes are significantly larger for programs that operated before 1980.)

Three other reviews (i.e., Camilli et al., 2010; Gorey, 2001; and Nelson et al., 2003) use study quality rating as a moderator in their analyses. These studies find, overall, little-to-no evidence that study quality is related to the size of effects. Of these reviews, two find no statistical relationship between effect size and study quality. Camilli et al. (2010), on the other hand, find some evidence that high quality studies “yielded larger effect sizes (about .27 ES)” (p. 599). Of the three domains studied, however, the effect was statistically significant only on the cognitive domain for the treatment versus no treatment contrast group and on the “social” domain for the treatment versus alternative contrast group. (There was no statistically significant difference for either contrast group for the “school” domain.)

**APPENDIX C**

**Non-Systematic Reviews Not Included in Appendix B, “Findings from Thirteen Systematic Reviews”**

| **Review** | **Reason for exclusion** |
| --- | --- |
| Anderson et al. (2003) | Did not explain how study quality was assessed. |
| Boocock (1995) | Did not provide sufficient description of methodology. |
| Currie (2001) | Did not explain how study quality was assessed. |
| Darrow (2009) | Did not provide sufficient description of methodology. |
| Elango, García, Heckman, and Hojman (2015) | Did not provide sufficient description of methodology and includes studies of only eleven ECE programs. |
| Fryer (2017) | Limited eligible study designs to randomized control trials (RCTs) but did not examine the implementation of the RCT. |
| van Huizen and Plantenga (2018) | Limited eligible study designs to those using regression discontinuity designs, difference in differences, and instrumental variables but, as far as we can tell, did not examine the implementation of those designs. |
| Karoly, Kilburn, and Cannon (2005) | Excluded research that was not “formally published” (p. 31) and, for a number of outcomes, report positive effects “if a significant effect was found in any program evaluation follow-up. For example, if a significant effect was observed at age 5 but not at age 9 the outcome is listed in this table” (p. 57). This would mean that one positive effect in a given domain has more weight than an infinite number of null effects, thereby overstating the effectiveness of ECE programs on improving socio-emotional outcomes. |
| Magnuson and Waldfogel (2005) | Did not provide sufficient description of methodology. |
| Melhuish et al. (2015) | Did not provide sufficient description of methodology. |
| Mitchell, Wylie, and Carr (2008) | Did not explain how study quality was assessed. |
| Ulferts and Anders (2015) | Did not explain how study quality was assessed. |
| White (1985) | Did not provide sufficient description of methodology. |
| Yoshikawa (1995) | Did not explain how study quality was assessed. |

**APPENDIX D**

**Biographies of Advisory Committee Members**

**Jacob A. Klerman** is a principal associate in the Social & Economic Policy division at Abt Associates. His current efforts focus on leading roles in the design and analysis of large random assignment trials for the U.S. Department of Labor and the U.S. Department of Agriculture. Mr. Klerman has extensive experience with survey design and analysis, as well as non-experimental program evaluation, and he has done multiple analyses for the U.S. Department of Health and Human Services and the U.S. Department of Defense. Within Abt, he is the director of the Evaluation and Monitoring Methods Center and co-director of Abt's Development and Dissemination Grant Program. Outside of Abt, he is the editor of Evaluation Review, a leading academic evaluation methods journal.

**Rebecca A. Maynard** is the University Trustee Professor of Education and Social Policy in the Graduate School of Education at University of Pennsylvania. She is a leading expert in the design and conduct of randomized controlled trials in the areas of education and social policy, has conducted influential methodological research, and recently published open-ware tools to support the efficient design of rigorous impact evaluations. Dr. Maynard has been a leader in the development and application of methods for conducting systematic reviews of evidence on program effectiveness, including serving on the technical review team during the design and development of the What Works Clearinghouse and being active in the workgroup that laid the groundwork for the Campbell Collaboration.

**Mike Puma** is the president of Chesapeake Research Associates. He has over 35 years of experience in program evaluation having conducted major national studies in K-12 and preschool education, nutrition assistance, income security, and employment and training. His current research activities include serving as the Principal Investigator for the 10-year National Head Start Impact Study (a national randomized experiment involving longitudinal study of about 4,000 preschool children) and Senior Researcher on several studies for the Institute for Educational Sciences including the Analytical and Technical Support contract, the experimental evaluation of the DC school voucher program, the What Works Clearinghouse, the study of highly selective alternative teacher certification programs, and the evaluation of the Teacher Incentive Fund.

**Matthew Stagner** is a senior fellow and Director of Human Services Research at Mathematica Policy Research (Chicago office). He is a nationally known expert on youth development and risk behaviors, child welfare, evaluation design and methods and the role of research in policymaking. Prior to joining Mathematica, he served as executive director of Chapin Hall and as a senior lecturer at the Irving B. Harris School of Public Policy Studies, both housed at the University of Chicago.

**APPENDIX E**

**Draft Coding Instrument (v. 19_1114**

| **Reference Information**  **(either enough information to make an APA citation or an actual APA citation)** | | | | | | | |  |
| --- | --- | --- | --- | --- | --- | --- | --- | --- |
| Title: | |  |  |  |  |  |  | |
| Authors: | |  |  |  |  |  |  | |
| Publication year: | |  |  |  |  |  |  | |
| Name of the intervention studied: | | | |  |  |  |  | |
| Is this a follow-on study (have the same kids in the same intervention been previously evaluated?) | | | | | | | | |
|  |  |  | 1 - Yes |  |  |  |  | |
|  |  |  | 0 - No |  |  |  |  | |
|  |  |  | 99 - Cannot tell |  |  |  |  | |
| Study ID: ______________________ | | | |  |  |  |  | |
|  | File name convention: Program_First author (last name)_Title of paper_Year | | | | | |  | |
| **Criteria for Inclusion** | | | | | | | | |
| **Stage 1 (Abstract/Title)** | | | |  |  |  |  | |
| Study ID: ______________________ | | | | | | | | |
| Coder initials: __ __ __ | | | |  |  |  |  | |
| 1. | Is the study reported in English? | | |  |  |  |  | |
|  |  |  | 1 - Yes |  |  |  |  | |
|  |  |  | 0 - No |  |  |  |  | |
| 2. | Was the study published in or after 1960? | | | |  |  |  | |
|  |  |  | 1 - Yes |  |  |  |  | |
|  |  |  | 0 - No |  |  |  |  | |
|  |  |  | 99 - Cannot tell |  |  |  |  | |
| 3. | Does the study report primary research findings? | | | |  |  |  | |
|  |  |  | 1 - Yes |  |  |  |  | |
|  |  |  | 0 - No |  |  |  |  | |
|  |  |  | 99 - Cannot tell |  |  |  |  | |
| 4. | Does the study report at least one cognitive, socio-emotional, behavioral, or health outcome or impact? | | | | | | | |
|  |  |  | 1 - Yes |  |  |  |  | |
|  |  |  | 0 - No |  |  |  |  | |
|  |  |  | 99 - Cannot tell |  |  |  |  | |
| 5. | Does the study estimate the effect of children's participation in what appears to be a preschool-type program? | | | | | | | |
|  |  |  | 1 - Yes |  |  |  |  | |
|  |  |  | 0 - No |  |  |  |  | |
|  |  |  | 99 - Cannot tell |  |  |  |  | |
| 6. | Did the program provide educational instruction directly to children? | | | | | |  | |
|  |  |  | 1 - Yes |  |  |  |  | |
|  |  |  | 0 - No |  |  |  |  | |
|  |  |  | 99 - Cannot tell |  |  |  |  | |
| 7. | Were children selected for participation because of a severe developmental disability, learning disability, developmental delay, or chronic medical disorder? | | | | | | | |
|  |  |  | 1 - Yes |  |  |  |  | |
|  |  |  | 0 - No |  |  |  |  | |
|  |  |  | 99 - Cannot tell |  |  |  |  | |
| 8. | Is there another apparent reason for excluding this study? (E.g., wrong age group, non-applicable country.) | | | | | | | |
|  |  |  | 1 - Yes |  |  |  |  | |
|  |  |  | 0 - No |  |  |  |  | |
|  |  |  | 99 - Cannot tell |  |  |  |  | |
| 9. | Is the study eligible for inclusion at this stage? | | | |  |  |  | |
|  |  |  | 1 - Yes |  |  |  |  | |
|  |  |  | 0 - No |  |  |  |  | |
|  |  |  | 99 - Cannot tell |  |  |  |  | |
|  |  |  |  |  |  |  |  | |
| **Stage 2 (Full text)** | | | | | | | | |
| Study ID: ______________________ | | | | | | | | |
| Coder initials: __ __ __ | | | |  |  |  |  | |
| 1. | Was the study conducted in an eligible country? | | | |  |  |  | |
|  |  |  | 1 - Yes |  |  |  |  | |
|  |  |  | 0 - No |  |  |  |  | |
|  |  |  | 99 - Cannot tell |  |  |  |  | |
| 2. | Did the intervention target children from birth to age five? (Select “yes” for studies evaluating Head Start, Early Head Start, or state pre-K.) | | | | | | | |
|  |  |  | 1 - Yes |  |  |  |  | |
|  |  |  | 0 - No |  |  |  |  | |
|  |  |  | 99 - Cannot tell |  |  |  |  | |
| 3. | Are participants described as, or are effects reported separately for disadvantaged/low income/at risk children? (Select “yes” for studies evaluating Head Start or Early Head Start.) | | | | | | | |
|  |  |  | 1 - Yes |  |  |  |  | |
|  |  |  | 0 - No |  |  |  |  | |
|  |  |  | 99 - Cannot tell |  |  |  |  | |
| 4. | Did the program provide educational instruction to children on topics such as math, language, and science as a primary component of the intervention? (Select “yes” for studies evaluating Head Start, Early Head Start, or state pre-K.) | | | | | | | |
|  |  |  | 1 - Yes |  |  |  |  | |
|  |  |  | 0 - No |  |  |  |  | |
|  |  |  | 99 - Cannot tell |  |  |  |  | |
| 5. | Did the program provide only supplemental instruction or tutorial services to children? | | | | | |  | |
|  |  |  | 1 - Yes |  |  |  |  | |
|  |  |  | 0 - No |  |  |  |  | |
|  |  |  | 99 - Cannot tell |  |  |  |  | |
| 6. | Does the study use student outcomes to evaluate the effectiveness of teacher credentials (or other characteristics), professional development programs for teachers, pedagogical approaches, or curriculum? (These are often compared to business-as-usual or other variations/alternatives.) | | | | | | | |
|  |  |  | 1 - Yes |  |  |  |  | |
|  |  |  | 0 - No |  |  |  |  | |
|  |  |  | 99 - Cannot tell |  |  |  |  | |
| 7. | Did the intervention occur primarily in a public school, center (public or private), or faith-based setting (church or other religious institution)? (Select “yes” for studies evaluating Head Start, Early Head Start, or state pre-K.) | | | | | | | |
|  |  |  | 1 - Yes |  |  |  |  | |
|  |  |  | 0 - No |  |  |  |  | |
|  |  |  | 99 - Cannot tell |  |  |  |  | |
| 8. | Did the program operate for at least one academic year (approximately 180 days)? (Select “yes” for studies evaluating Head Start/Early Head Start conducted after the Fall of 1965, or state pre-K.) | | | | | | | |
|  |  |  | 1 - Yes |  |  |  |  | |
|  |  |  | 0 - No |  |  |  |  | |
|  |  |  | 99 - Cannot tell |  |  |  |  | |
| 9. | Does the study report at least one immediate outcome or is it a follow-on to one that did so? | | | | | | | |
|  |  |  | 1 - Yes |  |  |  |  | |
|  |  |  | 0 - No |  |  |  |  | |
|  |  |  | 99 - Cannot tell |  |  |  |  | |
| 10. | Does the study employ either a comparison group, comparison time series, or control variable? | | | | | | | |
|  |  |  | 1 - Yes |  |  |  |  | |
|  |  |  | 0 - No |  |  |  |  | |
|  |  |  | 99 - Cannot tell |  |  |  |  | |
| 11. | Is the study eligible for inclusion at this stage? | | | |  |  |  | |
|  |  |  | 1 - Yes |  |  |  |  | |
|  |  |  | 0 - No |  |  |  |  | |
|  |  |  | 99 - Cannot tell |  |  |  |  | |
|  |  |  |  |  |  |  |  | |
| **Risk of bias/Study Rating Tool** | | | | | | | | |
| Study ID: ______________________ | | | | | | | | |
| Coder initials: __ __ __ | | | | | | | | |
| **Standard 1: Selection** | | | | | | | | |
| 1. | Assignment to condition | | |  |  |  |  | |
|  |  |  | 1 - Random assignment (no issues) | | | | | |
|  |  |  | 2 - Random assignment (issues reported) | | | |  | |
|  |  |  | 3 - Matched groups (*ex ante*) | | | | | |
|  |  |  | 4 - Match groups (*post hoc*) | | | | | |
|  |  |  | 5 - Propensity score matching | | | | | |
|  |  |  | 6 - Instrumental variable (or forcing variable, cut-off) | | | |  | |
|  |  |  | 7 - None of the above | |  |  |  | |
|  |  |  | 8 - Not applicable (comparison-to-self design) | | | |  | |
|  |  |  | 99 - Cannot tell |  |  |  |  | |
| 2. | Baseline equivalence | | |  |  |  |  | |
|  |  |  | 1 - Test scores |  |  |  |  | |
|  |  |  | 2 - Demographic characteristics | | |  |  | |
|  |  |  | 3 - Both test scores and demographic characteristics | | | |  | |
|  |  |  | 4 - Propensity score matching | | | | | |
|  |  |  | 5 - Fixed effects |  |  |  |  | |
|  |  |  | 6 - Difference-in-differences | | | | | |
|  |  |  | 7 - No equivalence | | | | | |
|  |  |  | 8 - Not applicable (comparison-to-self design) | | | |  | |
|  |  |  | 99 - Cannot tell |  |  |  |  | |
| 3. | Does this study satisfy standard 1? | | |  |  |  |  | |
|  |  |  | 1 - Yes |  |  |  |  | |
|  |  |  | 2 - Yes, *with reservations* | |  |  |  | |
|  |  |  | 3 - No |  |  |  |  | |
|  |  |  | 8 - Not applicable (comparison-to-self design) | | | |  | |
|  |  |  | 99 - Cannot tell |  |  |  |  | |
|  |  |  |  |  |  |  |  | |
| **Standard 2: Attrition** | | |  |  |  |  |  | |
| 1. | Does the study report attrition information for both the program and non-program group, if applicable? | | | | | | | |
|  |  |  | 1 - Yes |  |  |  |  | |
|  |  |  | 0 - No |  |  |  |  | |
|  |  |  | 3 - Age cutoff regression discontinuity design | | | |  | |
| 2. | Does attrition exceed the conservative boundaries in the WWC attrition standards? | | | | | |  | |
|  |  |  | 1 - Yes |  |  |  |  | |
|  |  |  | 0 - No |  |  |  |  | |
|  |  |  | 99 - Cannot tell |  |  |  |  | |
| 3. | Does this study satisfy standard 1? | | |  |  |  |  | |
|  |  |  | 1 - Yes |  |  |  |  | |
|  |  |  | 0 - No |  |  |  |  | |
|  |  |  | 99 - Cannot tell |  |  |  |  | |
|  |  |  |  |  |  |  |  | |
| **Full Study Coding** | | | | | | | | |
| Study ID: ______________________ | | | | | | | | |
| Coder initials: __ __ __ | | | |  |  |  |  | |
|  |  |  |  |  |  |  |  | |
| **Study characteristics** | | |  |  |  |  |  | |
| 1. | Type of report: | |  |  |  |  |  | |
|  |  |  | 1 - Journal article | |  | 6 - Government report |  | |
|  |  |  | 2 - Book or book chapter | |  | 7 - Conference paper |  | |
|  |  |  | 3 - Dissertation |  |  | 9 - Other (Specify): _______________ | | |
|  |  |  | 4 - MA Thesis |  |  | 99 - Cannot tell | | |
|  |  |  | 5 - Private report | |  |  |  | |
| 2. | Has the report been subject to peer review? | | | |  |  |  | |
|  |  |  | 1 - Yes |  |  |  |  | |
|  |  |  | 0 - No |  |  |  |  | |
|  |  |  | 99 - Cannot tell |  |  |  |  | |
| 3. | How was the study found? | | |  |  |  |  | |
|  |  |  | 1 - Electronic database (Specify: ____________________) | | | |  | |
|  |  |  | 2 - Website - research firm | |  |  |  | |
|  |  |  | 3 - Website - gov't agency | | | |  | |
|  |  |  | 4 - Website - association or advocacy group | | | |  | |
|  |  |  | 5 - Hand search |  |  |  |  | |
|  |  |  | 6 - Reference in a study/book | | | |  | |
|  |  |  | 7 - International trial register | | | |  | |
|  |  |  | 8 - Duncan and Magnuson's database | | | |  | |
|  |  |  | 9 - Other |  |  |  |  | |
| 4. | Companion studies: | | |  |  |  |  | |
|  |  | ID # | _____________________ | |  |  |  | |
|  |  | ID # | _____________________ | |  |  |  | |
|  |  | ID # | _____________________ | |  |  |  | |
| 5. | Was the evaluator involved in program operation? | | | | |  |  | |
|  |  |  | 1 - Yes |  |  |  |  | |
|  |  |  | 0 - No |  |  |  |  | |
|  |  |  | 99 - Cannot tell |  |  |  |  | |
| 6. | Where was the report published? | | |  |  |  |  | |
|  |  |  | 1 - US |  |  |  |  | |
|  |  |  | 2 - Europe |  |  |  |  | |
|  |  |  | 3 - South America | | | |  | |
|  |  |  | 4 - OECD |  |  |  |  | |
|  |  |  | 5 - Oceania | | | |  | |
|  |  |  | 6 - Canada | | | |  | |
|  |  |  | 9 - Other (Specify: ________________) | | | |  | |
| 7. | Where was the study conducted? | | |  |  |  |  | |
|  |  |  | 1 - US |  |  |  |  | |
|  |  |  | 2 - Europe |  |  |  |  | |
|  |  |  | 3 - South America | | | |  | |
|  |  |  | 4 - OECD |  |  |  |  | |
|  |  |  | 5 - Oceania | | | |  | |
|  |  |  | 6 - Canada | | | |  | |
|  |  |  | 9 - Other (Specify: ________________) | | | |  | |
| 8. | Study sponsorship or funding: | | |  |  |  |  | |
|  |  |  | 1 - Federal |  |  |  |  | |
|  |  |  | 2 - State/province | | | |  | |
|  |  |  | 3 - Local/school district | |  |  |  | |
|  |  |  | 4 - University |  |  |  |  | |
|  |  |  | 5 - Non-profit/foundation | |  |  |  | |
|  |  |  | 6 - Faith-based funding | |  |  |  | |
|  |  |  | 9 - Other |  |  |  |  | |
|  |  |  | 99 - Cannot tell |  |  |  |  | |
| 9. | Year study was reported: | | |  |  |  |  | |
|  |  |  | __________ |  |  |  |  | |
|  |  |  | 99 - Cannot tell |  |  |  |  | |
| 10. | Year study was started: | | |  |  |  |  | |
|  |  |  | __________ |  |  |  |  | |
|  |  |  | 99 - Cannot tell |  |  |  |  | |
| 11. | Year study was completed: | | |  |  |  |  | |
|  |  |  | __________ |  |  |  |  | |
|  |  |  | 99 - Cannot tell |  |  |  |  | |
| ***Population*** | |  |  |  |  |  |  | |
| 12. | Is the program available to all age- and location-appropriate children? (Is it a universal program?) | | | | | | | |
|  |  |  | 1 - Yes |  |  |  |  | |
|  |  |  | 0 - No |  |  |  |  | |
|  |  |  | 99 - Cannot tell |  |  |  |  | |
| 13. | What was the predominant (> 50%) economic background of program participants? | | | | | |  | |
|  |  |  | 1 - Low-income |  |  |  |  | |
|  |  |  | 2 - Middle/ high income | |  |  |  | |
|  |  |  | 3 - Diverse SES |  |  |  |  | |
|  |  |  | 99 - Cannot tell |  |  |  |  | |
| 14. | What percent of study participants were disadvantaged/low income? | | | | | |  | |
|  |  |  | __________ |  |  |  |  | |
|  |  |  | 99 - Cannot tell |  |  |  |  | |
| 15. | What percent of children were English Language Learners? | | | | | |  | |
|  |  |  | __________ |  |  |  |  | |
|  |  |  | 99 - Cannot tell |  |  |  |  | |
| 16. | What was the racial composition of the *full sample*? | | | | |  |  | |
|  |  |  | 1 - White | ______ |  |  |  | |
|  |  |  | 2 - Black | ______ |  |  |  | |
|  |  |  | 3 - Hispanic | ______ |  |  |  | |
|  |  |  | 9 - Other | ______ |  |  |  | |
|  |  |  | 99 - Cannot tell |  |  |  |  | |
| 17. | What was the racial composition of the *intervention* group? | | | | | |  | |
|  |  |  | 1 - White | ______ |  |  |  | |
|  |  |  | 2 - Black | ______ |  |  |  | |
|  |  |  | 3 - Hispanic | ______ |  |  |  | |
|  |  |  | 9 - Other | ______ |  |  |  | |
|  |  |  | 99 - Cannot tell |  |  |  |  | |
| 18. | What was the racial composition of the *non-program* group? | | | | | |  | |
|  |  |  | 1 - White | ______ |  |  |  | |
|  |  |  | 2 - Black | ______ |  |  |  | |
|  |  |  | 3 - Hispanic | ______ |  |  |  | |
|  |  |  | 7- N/A, no non-program group | | |  |  | |
|  |  |  | 9 - Other | ______ |  |  |  | |
|  |  |  | 99 - Cannot tell |  |  |  |  | |
| 19. | How old were children when receiving services? (Check all that apply) | | | | | |  | |
|  |  |  | 1 - Less than 1 |  |  |  |  | |
|  |  |  | 2 - 1-2 |  |  |  |  | |
|  |  |  | 3 - 2-3 |  |  |  |  | |
|  |  |  | 4 - 3-4 |  |  |  |  | |
|  |  |  | 5 - 4-5 |  |  |  |  | |
|  |  |  | 9 - Other |  |  |  |  | |
|  |  |  | 99 - Cannot tell |  |  |  |  | |
| 20. | What was the gender composition of the *full sample*? | | | | |  |  | |
|  |  |  | 1 - % Female  _______ | (use 99 if cannot tell) | | |  | |
| 21. | What was the gender composition of the *intervention* group? | | | | | |  | |
|  |  |  | 1 - % Female  _______ | (use 99 if cannot tell) | | |  | |
| 22. | What was the gender composition of the *non-program group*? | | | | | |  | |
|  |  |  | 1 - % Female  _______ | (use 99 if cannot tell) | | |  | |
| 23. | Were participating children drawn from a nationwide sample? | | | | | |  | |
|  |  |  | 1 - Yes |  |  |  |  | |
|  |  |  | 0 - No |  |  |  |  | |
|  |  |  | 99 - Cannot tell |  |  |  |  | |
|  |  |  |  |  |  |  |  | |
| ***Intervention*** | |  |  |  |  |  |  | |
| 24. | How long did the program operate? | | | |  |  |  | |
|  |  |  | 1 - More than 1 year | |  |  |  | |
|  |  |  | 2 - One calendar year | |  |  |  | |
|  |  |  | 3 - Academic year |  |  |  |  | |
|  |  |  | 9 - Other |  |  |  |  | |
|  |  |  | 99 - Cannot tell |  |  |  |  | |
| 25. | Did the program use a curriculum? | | |  |  |  |  | |
|  |  |  | 1 - Yes |  |  |  |  | |
|  |  |  | 0 - No |  |  |  |  | |
|  |  |  | 99 - Cannot tell |  |  |  |  | |
| 26. | If yes, which curriculum was used? | | |  |  |  |  | |
|  |  |  | 1 - Creative Curriculum | |  |  |  | |
|  |  |  | 2 - DLM Early Childhood Express | | | |  | |
|  |  |  | 3 - HighReach Learning | |  |  |  | |
|  |  |  | 4 - HighScope |  |  |  |  | |
|  |  |  | 5 - Montessori |  |  |  |  | |
|  |  |  | 6 - Scholastic Big Day for Pre-k | | |  |  | |
|  |  |  | 9 - Other (Specify: ______________) | | | |  | |
|  |  |  | 99 - Cannot tell |  |  |  |  | |
| 27. | What was the predominant level of certification that teachers possessed? | | | | | |  | |
|  |  |  | 1 - Associates degree | |  |  |  | |
|  |  |  | 2 - Bachelor's degree | |  |  |  | |
|  |  |  | 3 - Master's degree | |  |  |  | |
|  |  |  | 4 - ECE certificate | | | |  | |
|  |  |  | 5 - ECE certificate *and* degree | | |  |  | |
|  |  |  | 6 - No requirement | | | |  | |
|  |  |  | 9 - Other |  |  |  |  | |
|  |  |  | 99 - Cannot tell |  |  |  |  | |
| 28. | How many hours per day did the program operate? | | | | |  |  | |
|  |  |  | 1 - Half-day (2 1/2 to 3 hours) | | |  |  | |
|  |  |  | 2 - Full day (6 hours) | |  |  |  | |
|  |  |  | 9 - Other |  |  |  |  | |
|  |  |  | 99 - Cannot tell |  |  |  |  | |
| 29. | What was the primary setting of the program? | | | |  |  |  | |
|  |  |  | 1 - Public school |  |  |  |  | |
|  |  |  | 2 - Private center | | | |  | |
|  |  |  | 3 - Public center |  |  |  |  | |
|  |  |  | 4 - Faith-based center | |  |  |  | |
|  |  |  | 9 - Other |  |  |  |  | |
|  |  |  | 99 - Cannot tell |  |  |  |  | |
| 30. | Was this a demonstration project or a one-off, or an on-going program? | | | | | |  | |
|  |  |  | 1 - Yes |  |  |  |  | |
|  |  |  | 0 - No |  |  |  |  | |
|  |  |  | 99 - Cannot tell |  |  |  |  | |
| 31. | Primary location of the program: | | |  |  |  |  | |
|  |  |  | 1 - Urban area |  |  |  |  | |
|  |  |  | 2 - Suburban area | | | |  | |
|  |  |  | 3 - Rural area |  |  |  |  | |
|  |  |  | 4 - A mixture of areas | |  |  |  | |
|  |  |  | 99 - Cannot tell |  |  |  |  | |
| 32. | Did the program provide ancillary services to children and/or parents/caregivers? | | | | | |  | |
|  |  |  | 1 - Yes |  |  |  |  | |
|  |  |  | 0 - No |  |  |  |  | |
|  |  |  | 99 - Cannot tell |  |  |  |  | |
| 33. | If yes, what ancillary services were provided? | | | |  |  |  | |
|  |  |  | 1 - Health services to children | | | |  | |
|  |  |  | 2 - Health services to families | | | |  | |
|  |  |  | 3 - Counseling services to children | | | |  | |
|  |  |  | 4 - Counseling services to families | | | |  | |
|  |  |  | 5 - Parenting classes/instruction | | |  |  | |
|  |  |  | 6 - Employment counseling | | | |  | |
|  |  |  | 7 - No ancillary services | |  |  |  | |
|  |  |  | 9 - Other (Specify: ____________________) | | | |  | |
|  |  |  | 99 - Cannot tell |  |  |  |  | |
| 34. | What was the average class size/group size in the program? | | | | | |  | |
|  |  |  | _____ per _____ adults | |  |  |  | |
|  |  |  | 99 - Cannot tell |  |  |  |  | |
| 35. | What are the sources of funding of the interventions | | | | |  |  | |
|  |  |  | 1 - Federal |  |  |  |  | |
|  |  |  | 2 - State/province | | | |  | |
|  |  |  | 3 - Local/school district | |  |  |  | |
|  |  |  | 4 - University |  |  |  |  | |
|  |  |  | 5 - Non-profit/foundation | |  |  |  | |
|  |  |  | 6 - Faith-based funding | |  |  |  | |
|  |  |  | 9 - Other |  |  |  |  | |
|  |  |  | 99 - Cannot tell |  |  |  |  | |
| 36. | What organization had the primary responsibility for operating and maintaining this intervention? | | | | | | | |
|  |  |  | 1 - University |  |  |  |  | |
|  |  |  | 2 - School/school district | |  |  |  | |
|  |  |  | 3 - Social services organization | | |  |  | |
|  |  |  | 4 - Community-based organization/non-profit | | | |  | |
|  |  |  | 5 - Faith-based organization | | | |  | |
|  |  |  | 9 - Other |  |  |  |  | |
|  |  |  | 99 - Cannot tell |  |  |  |  | |
| 37. | If collaborative, what organization(s) had secondary responsibility? | | | | | |  | |
|  |  |  | 1 - University/college research center | | | |  | |
|  |  |  | 2 - School/school district | |  |  |  | |
|  |  |  | 3 - Social services organization | | |  |  | |
|  |  |  | 4 - Community-based organization/non-profit | | | |  | |
|  |  |  | 5 - Faith-based organization | | | |  | |
|  |  |  | 9 - Other |  |  |  |  | |
|  |  |  | 99 - Cannot tell |  |  |  |  | |
|  |  |  | 999 - Not collaborative | |  |  |  | |
| 38. | How much did the program cost per student? | | | |  |  |  | |
|  |  |  | $ ___________ |  |  |  |  | |
|  |  |  | 99 - Cannot tell |  |  |  |  | |
|  |  |  |  |  |  |  |  | |
| ***Outcomes*** | |  |  |  |  |  |  | |
| 39. | How many outcomes were measured? | | | |  |  |  | |
|  |  |  | ___________ |  |  |  |  | |
|  |  |  | 99 - Cannot tell |  |  |  |  | |
| 40. | How many outcomes were reported? | | |  |  |  |  | |
|  |  |  | ___________ |  |  |  |  | |
|  |  |  | 99 - Cannot tell |  |  |  |  | |
| 41. | For which outcome domains were results reported? | | | | |  |  | |
|  |  |  | 1 - Cognitive |  |  |  |  | |
|  |  |  | 2 - Socioemotional | | | |  | |
|  |  |  | 3 - Behavioral |  |  |  |  | |
|  |  |  | 4 - Health |  |  |  |  | |
|  |  |  | 9 - Other |  |  |  |  | |
|  |  |  |  |  |  |  |  | |
|  | Fill in Data sheet for each eligible outcome | | | |  |  |  | |
|  |  |  |  |  |  |  |  | |
| **Study Design** | |  |  |  |  |  |  | |
| 42. | Was the evaluation an experiment or an observational study? | | | | | |  | |
|  |  |  | 1 - Experiment |  |  |  |  | |
|  |  |  | 2 - Observational study | |  |  |  | |
|  |  |  | 99 - Cannot tell |  |  |  |  | |
| 43. | Did the study employ a two-group design (both a program and non-program group)? | | | | | |  | |
|  |  |  | 1 - Yes |  |  |  |  | |
|  |  |  | 0 - No |  |  |  |  | |
|  |  |  | 99 - Cannot tell |  |  |  |  | |
| 44. | How were children assigned to the condition? | | | |  |  |  | |
|  |  |  | 1 - Random, simple | |  |  |  | |
|  |  |  | 2 - Random after matching, stratification, etc. | | | |  | |
|  |  |  | 3 - *Ex ante* matching | |  |  |  | |
|  |  |  | 4 - *Post hoc* matching | |  |  |  | |
|  |  |  | 5 - Propensity score matching | | | |  | |
|  |  |  | 6 - Time series/cohort comparison | | | |  | |
|  |  |  | 9 - Nonrandom, other | |  |  |  | |
|  |  |  | 99 - Cannot tell |  |  |  |  | |
| 45. | What level of services did the control/comparison group receive? | | | | | |  | |
|  |  |  | 1 - None |  |  |  |  | |
|  |  |  | 2 - Some, participants enrolled in other similar programs | | | |  | |
|  |  |  | 3 - Some, received services as part of study design | | | |  | |
|  |  |  | 4 - Some, participants received services from the intervention not according to study design | | | | | |
|  |  |  | 99 - Cannot tell |  |  |  |  | |
| 46. | Unit of assignment to treatment condition | | | |  |  |  | |
|  |  |  | 1 - Individual child | | | |  | |
|  |  |  | 2 - Group |  |  |  |  | |
|  |  |  | 3 - Classroom |  |  |  |  | |
|  |  |  | 4 - School |  |  |  |  | |
|  |  |  | 5 - Region, community, school district, etc. | | | |  | |
|  |  |  | 9 - Other |  |  |  |  | |
|  |  |  | 99 - Cannot tell |  |  |  |  | |
| 47. | Unit of assignment to control/comparison group? | | | | | |  | |
|  |  |  | 1 - Individual child | | | |  | |
|  |  |  | 2 - Group |  |  |  |  | |
|  |  |  | 3 - Classroom |  |  |  |  | |
|  |  |  | 4 - School |  |  |  |  | |
|  |  |  | 5 - Region, community, school district, etc. | | | |  | |
|  |  |  | 6 - N/A, no control/comparison group | | | |  | |
|  |  |  | 9 - Other |  |  |  |  | |
|  |  |  | 99 - Cannot tell |  |  |  |  | |
| 48. | Was the data collector blind to the group assignment? | | | | | | | |
|  |  |  | 1 - Yes |  |  |  |  | |
|  |  |  | 0 - No |  |  |  |  | |
|  |  |  | 99 - Cannot tell |  |  |  |  | |
| 49. | What is the risk of substitution bias? | | | | | |  | |
|  |  |  | 1 - Low (< 25%) |  |  |  |  | |
|  |  |  | 2 - Medium (25-75%) | |  |  |  | |
|  |  |  | 3 - High (> 75%) | | | |  | |
|  |  |  | 99 - Cannot tell |  |  |  |  | |
| 50. | What is the risk of crossover bias? | | |  |  |  |  | |
|  |  |  | 1 - Low |  |  |  |  | |
|  |  |  | 2 - Medium |  |  |  |  | |
|  |  |  | 3 - High |  |  |  |  | |
|  |  |  | 99 - Cannot tell |  |  |  |  | |
| 51. | What is the risk of diffusion of treatment? | | | |  |  |  | |
|  |  |  | 1 - Low |  |  |  |  | |
|  |  |  | 2 - Medium |  |  |  |  | |
|  |  |  | 3 - High |  |  |  |  | |
|  |  |  | 99 - Cannot tell |  |  |  |  | |
| 52. | What is the risk of contagion? | | |  |  |  |  | |
|  |  |  | 1 - Low |  |  |  |  | |
|  |  |  | 2 - Medium |  |  |  |  | |
|  |  |  | 3 - High |  |  |  |  | |
|  |  |  | 99 - Cannot tell |  |  |  |  | |
| 53. | What is the risk of overall contamination? | | | |  |  |  | |
|  |  |  | 1 - Low (threat is low for all four of the above) | | | |  | |
|  |  |  | 2 - Medium (threat is medium for ≤ 2 of the above; low for others) | | | | | |
|  |  |  | 3 - High (threat is high for 1 of the above or medium for > 2) | | | |  | |
|  |  |  | 99 - Cannot tell |  |  |  |  | |
| 54. | Was the program implemented with fidelity to its design? | | | | | |  | |
|  |  |  | 1 - Yes |  |  |  |  | |
|  |  |  | 0 - No |  |  |  |  | |
|  |  |  | 99 - Cannot tell |  |  |  |  | |

1. As described above (see “limitations of these previous reviews”), this is a very broad domain that may encourage the combining of dissimilar outcomes (the “apples and oranges” problem). The WWC uses, seven outcome domains for what might be broadly classified as cognition in ECE research: (1) cognition (general), (2) mathematics, (3) language development, (4) alphabetics, (5) fluency, (6) comprehension, and (7) general reading achievement. We intend to use these domain classifications, to the extent that we can. [↑](#footnote-ref-1)
2. It should be noted that Burger’s (2010) systematic review “largely omits” studies that were included in earlier reviews “like the ones by Anderson (2003), Barnett (1995), Boocock (1995), and Currie (2001)” (pp. 143-144). This is a possible explanation for the little evidence they found for the effects of ECE participation on grade retention, special education placement, and high school graduation. It also may indicate that the effects on grade retention and placement in special education are largely driven by these older studies, which may, therefore, suggest that the findings in these older studies are not generalizable to today’s environment. [↑](#footnote-ref-2)
3. ^1^D’Onise et al. (2010a) and D’Onise et al. (2010b) do not report statistical significance; instead, only providing 95 percent confidence intervals for each effect size, risk ratio, and absolute risk difference. We infer statistical significance by whether or not the confidence interval includes zero. [↑](#footnote-ref-3)
4. D’Onise et al. (2010b) state that they take into account “clinical significance” but do not explicitly indicate which findings are such. Given this statement and that the “direction of association” findings in their Table 4 do not seem to be based on whether the confidence interval includes zero, we presume, that the designation as beneficial, adverse, or no difference is based on clinical significance. [↑](#footnote-ref-4)
5. Although D’Onise et al. (2010a) report a “suspensions/crime” outcome, in which they combine measures of both outcomes, we do not discuss this findings here because we consider these outcomes to be dissimilar. [↑](#footnote-ref-5)
6. Gorey (2001) finds that ECE participants are 59 percent less likely to have “ever engaged in delinquent behavior,” 53 percent less likely to have ever been arrested, and 82 percent less likely to have been “arrested five or more times” (p. 21), although these effects are based on three, four, and one effect sizes, respectively. Manning et al. (2010) find an effect size of 0.24, which is also statistically significant (p < 0.01). This effect is based on five effect sizes. [↑](#footnote-ref-6)
